# Dynamic optimization of biological networks under parametric uncertainty

**DOI:** 10.1186/s12918-016-0328-6

**Published:** 2016-08-31

**Authors:** Philippe Nimmegeers, Dries Telen, Filip Logist, Jan Van Impe

**Affiliations:** KU Leuven, Department of Chemical Engineering, BioTeC+ & OPTEC, Gebroeders De Smetstraat 1, Ghent, 9000 Belgium

**Keywords:** Dynamic optimization, Optimization under uncertainty, Biological networks, Multi-objective

## Abstract

**Background:**

Micro-organisms play an important role in various industrial sectors (including biochemical, food and pharmaceutical industries). A profound insight in the biochemical reactions inside micro-organisms enables an improved biochemical process control. Biological networks are an important tool in systems biology for incorporating microscopic level knowledge. Biochemical processes are typically dynamic and the cells have often more than one objective which are typically conflicting, e.g., minimizing the energy consumption while maximizing the production of a specific metabolite. Therefore multi-objective optimization is needed to compute trade-offs between those conflicting objectives. In model-based optimization, one of the inherent problems is the presence of uncertainty. In biological processes, this uncertainty can be present due to, e.g., inherent biological variability. Not taking this uncertainty into account, possibly leads to the violation of constraints and erroneous estimates of the actual objective function(s). To account for the variance in model predictions and compute a prediction interval, this uncertainty should be taken into account during process optimization. This leads to a challenging optimization problem under uncertainty, which requires a robustified solution.

**Results:**

Three techniques for uncertainty propagation: linearization, sigma points and polynomial chaos expansion, are compared for the dynamic optimization of biological networks under parametric uncertainty. These approaches are compared in two case studies: *(i)* a three-step linear pathway model in which the accumulation of intermediate metabolites has to be minimized and *(ii)* a glycolysis inspired network model in which a multi-objective optimization problem is considered, being the minimization of the enzymatic cost and the minimization of the end time before reaching a minimum extracellular metabolite concentration. A Monte Carlo simulation procedure has been applied for the assessment of the constraint violations. For the multi-objective case study one Pareto point has been considered for the assessment of the constraint violations. However, this analysis can be performed for any Pareto point.

**Conclusions:**

The different uncertainty propagation strategies each offer a robustified solution under parametric uncertainty. When making the trade-off between computation time and the robustness of the obtained profiles, the sigma points and polynomial chaos expansion strategies score better in reducing the percentage of constraint violations. This has been investigated for a normal and a uniform parametric uncertainty distribution. The polynomial chaos expansion approach allows to directly take prior knowledge of the parametric uncertainty distribution into account.

**Electronic supplementary material:**

The online version of this article (doi:10.1186/s12918-016-0328-6) contains supplementary material, which is available to authorized users.

## Background

The application of micro-organisms in chemical industry and life sciences is paramount. In industrial biotechnology, on the one hand, microbial growth is stimulated in order to enhance the production of (high added value) chemical and pharmaceutical products. On the other hand, in food industry the aim is to avoid the growth of pathogens and food spoilage to ensure food safety.

Therefore, a profound biochemical insight in microbial dynamics and the reactions inside micro-organisms is important. Integrating insights obtained at systems biology (microscopic) level contributes to an improved (macroscopic level) biochemical process control (i.e., enabling advanced model based monitoring, control and optimization of bioprocesses) [1].

A basic tool in systems biology for incorporating microscopic level information are biological networks, e.g., metabolic reaction networks in which the knots represent the metabolites (chemical substances produced/consumed in the micro-organisms) and the connections indicate the mass fluxes between those metabolites. A biological network is a systematic representation of the cellular processes and the interactions between the molecules in the cells: e.g., proteins and metabolites. Such a network comprises (a subset of) all reactions which occur inside a cell and the knots represent the metabolites (i.e., products consumed/produced by the cells) and the links represent the intracellular reactions or reactions between the cell and its environment. A cell can be seen on microscopic scale as a combination of interactions between different layers: fluxome, metabolome, proteome, transcriptome and genome. In terms of network complexity (i.e., the number of metabolites and fluxes), fluxome level biological networks have the lowest level of complexity, while genome-scale biological networks have the highest level of complexity [2–4].

Insight in the dynamic behavior of micro-organisms can be obtained by simulation of metabolic networks. Optimization of biological networks can be used to analyze and also influence the regulation of pathways, e.g., to stimulate the production of high added value products. In practice, cells often have more than one objective, which are conflicting, e.g., minimizing the energy consumption while maximizing the production of a certain metabolite. Therefore, dynamic (multi-objective) optimization, which provides optimal (possibly time-varying) control profiles, is an important tool. Multi-objective optimization of biological networks has been investigated in [5–7]. The multi-objective design of bioprocesses and solution strategies have for instance been presented in [8] with application to a well-stirred, aerobic fermentor in which *Saccharomyces cerevisiae* grows in a medium of sugar cane molasses.

However, in practice, uncertainty on the model parameters and external process disturbances are inherently present. Uncertainty can originate from unmodeled process variables (*process noise*), e.g., inherent biological variability between cells which are genetically identical [9] or from a parameter estimation procedure based on noisy measurements (*measurement noise*), such that the true parameter values (which are different from the model parameters) are unknown. Not taking this uncertainty into account, possibly leads to the violation of constraints and erroneous estimates of the actual objective function(s). Therefore, the information about the uncertainty has to be taken into account to obtain *robustified controls* (i.e., variables that can be manipulated throughout the process) that ensure that constraints are met and an overall better objective function estimate is guaranteed. In this work the nature of uncertainty is assumed to be *stochastic*, i.e., following a probability distribution, and the uncertainty is modeled in the model parameters, i.e., *parametric uncertainty* [10].

Including robustness in an optimization problem is often tedious, since this typically leads to semi-infinite optimization problems that are challenging to solve in practice [10]. Three methods are compared in this work to approximately solve the *(multi-objective) dynamic optimization problem under parametric uncertainty* for biological networks: linearization [11], sigma points [12] and polynomial chaos expansion [13, 14]. Each of these methods requires increasing levels of information on the parametric uncertainty distribution to propagate the parametric uncertainty towards the states, constraints or objectives of interest.

The authors want to highlight that enzyme activation in biological networks has been studied in terms of dynamic optimization, single objective as well as multi-objective. In this work, for the first time, parametric uncertainty is taken into account for prediction and control of biological networks. Another novelty is the critical comparison of the linearization, sigma points and polynomial chaos expansion approaches for dynamic optimization of biological networks under uncertainty. Single objective as well as multi-objective optimization case studies have been investigated in this work. Therefore the general formulations in this work have been presented for multi-objective optimization problems.

The paper is structured as follows. In the ‘Methods’ section the multi-objective dynamic optimization problem formulation under parametric uncertainty is first presented. Then the concept of uncertainty propagation is introduced, together with the three applied approximation techniques for uncertainty propagation. Subsequently, multi-objective optimization methods are briefly discussed. To conclude this section the software and case studies are presented. A validation and assessment of the approximation techniques for uncertainty propagation based on the case studies is presented in the ‘Results and discussion’ section, together with a physical/biological interpretation. Finally, the ‘Conclusions’ section summarizes the main results of this work.

## Methods

In this section the robustified multi-objective dynamic optimization formulation is presented. Subsequently, the different approximation techniques for uncertainty propagation that enable a robustified dynamic optimization under parametric uncertainty are discussed. Next, the approach for the Monte Carlo simulations is presented. In addition, multi-objective optimization methods are introduced, followed by a brief discussion on the software used in this work. To conclude the case studies are presented.

### Multi-objective dynamic optimization under parametric uncertainty

Consider the system $\dot {\mathbf {x}}= \mathbf {f}(\mathbf {x},\mathbf {u},\boldsymbol {\theta },t)$, with $\mathbf {x}\in \mathbb {R}^{n_{x}}$ the state vector (e.g., metabolite concentrations), $\mathbf {u}\in \mathbb {R}^{n_{u}}$, the control vector (e.g., enzyme expression rates), $\boldsymbol {\theta }\in \mathbb {R}^{n_{\theta }}$ the vector containing the uncertain parameters (e.g., kinetic constants such as the maximum reaction rate) and *t* the time. The aim of a multi-objective dynamic optimization problem is to design a control, which minimizes several objective functions $\{J_{1}, \dots, J_{n_{J}}\}$, subject to the constraints (i.e., model as dynamic constraint and other constraints). The multi-objective dynamic optimization problem in the time interval *t*∈[0,*t*_f_] and constraints $\mathbf {c}(\mathbf {x},\mathbf {u},\boldsymbol {\theta },t)\in \mathbb {R}^{n_{c}}$ (e.g., bounds on the metabolite concentrations or fluxes for cell viability) is formulated as in Eq. (1).

1 $$\begin{array}{*{20}l}  &\underset{\mathbf{u, x}, t_{\mathrm{f}}}{\text{min}} \quad \{ J_{1}, \dots, J_{n_{J}} \}\\ \text{s.t.} & \left\{ \begin{array}{lll} \dot{\mathbf{x}} &=& \mathbf{f}(\mathbf{x},\mathbf{u},\boldsymbol{\theta},t) \\ \mathbf{x}(0) &=& \mathbf{x}_{0} \\ \mathbf{0} &\geq& \mathbf{c}(\mathbf{x},\mathbf{u},\boldsymbol{\theta},t) \end{array} \right. \end{array} $$

An inherent problem in the modeling of biological processes is uncertainty. This uncertainty can originate from model uncertainty and external disturbances [10]. The emphasis in this work is on parametric uncertainty, i.e., the uncertainty is present in several model parameters, which for instance can originate from biological variability. Not taking this uncertainty into account can possibly lead to constraint violations or erroneous estimates of the actual objective function of the process. In the field of robust optimization these uncertainties are taken into account to guarantee that critical constraints are not violated [10].

If knowledge about the parametric uncertainty distribution is present, expected values for the states and chance constraints can be formulated [15]. Chance constraints express that the probability of a constraint to be valid must be larger than a specific value [16, 17].

Consider that the constraints **0**≥**c**(**x**,**u**,***θ***,*t*) can be replaced by $n_{c_{\text {prob}}}$ chance constraints *c*_prob,*i*_, expressing that the probability that a constraint is satisfied is larger than a preset probability *β*_*i*_, with $i = 1, \hdots, n_{c_{\text {prob}}}$. In this work only single chance constraints are considered.

2 $$\begin{array}{*{20}l}  \beta_{i} \leq \mathbf{Pr}\left[ \mathbf{0}\geq c_{\text{prob},i}(\mathbf{x}, \mathbf{u},\boldsymbol{\theta},t)\right] \end{array} $$

If the uncertainty is fully known within a specific bounded set, the optimization problem is solved for the *worst-case scenario* in which all constraints have to be satisfied [15]. This approach typically leads to minmax problems which are hard to solve [18]. Since the worst-case scenario is often highly unlikely to occur, this approach can lead to poor results [11]. In order to solve this, a trade-off between the nominal case (i.e., the non-robustified case in which uncertainty is not taken into account and the nominal parameter values are used) and worst-case scenario can be made [15].

The main limitation of the dynamic optimization problem with chance constraints is solving the problem in a computationally efficient way. The propagation of the parameter uncertainties through the nonlinear model and obtaining computationally tractable expressions for the dynamic optimization problem with chance constraints remains challenging [19]. Therefore, the chance constraints can be approximated by deterministic constraints as in Eq. (3).

3 $$\begin{array}{*{20}l}  0 \geq \mathbf{E}\left[c_{\mathrm{prob,i}}\right] + \alpha_{c_{\text{prob},i}} \sqrt{\mathbf{Var}\left[c_{\mathrm{prob,i}}\right]} \end{array} $$

In Eq. (3), **E**[*c*_prob,i_] and **V** **a** **r**[*c*_prob,i_] express the expected value and variance of the chance constraint function *c*_prob,i_, respectively. The coefficient $\alpha _{c_{\text {prob},i}}$ is introduced as a *backoff parameter* (e.g., [11, 20]) to take the uncertainty on the chance constraints into account. The choice of the backoff can for instance correspond to a probability that the specific constraint is violated, i.e., so-called *single chance constraints*.

A first way to choose the backoff parameter $\alpha _{c_{\text {prob},i}}$ is with Cantelli-Chebyshev’s inequality. In [17] it is shown that an upper bound for the expected value on an individual chance constraint can be calculated. This equation holds for any underlying distribution of the chance constraint. Computing the backoff parameter via Cantelli-Chebyshev’s inequality for a probability of 95 % for the chance constraint to be satisfied, results in a backoff parameter $\alpha _{c_{\text {prob},i}}=4.36$, while for a normal probability distribution the backoff parameter would be 1.96. From this, it is clear that Cantelli-Chebyshev’s inequality generally leads to a very conservative bound with too high backoff parameter values for use in practice, leading potentially to infeasibilities.

If a probability distribution is assumed for the considered constraint(s) or objective function(s), a second way is to choose the backoff parameter based on the quantiles. For this a procedure as in [10] can be followed to obtain a desired confidence level for the constraint to be satisfied or to cover the objective function in a prediction interval. In this work the choice of the backoff parameter is based on the quantiles, assuming that the states follow a normal distribution, as shown in Table 1.

**Table 1:** Backoff parameter *α*_*i*_ with corresponding quantiles and confidence levels

*α*_*i*_	0.84	1.28	1.65	1.96
Quantile	0.20	0.10	0.05	0.025
Confidence level	0.80	0.90	0.95	0.975

Similarly to the reformulation of constraints, the objective function *J*_*i*_ can be reformulated by adding the term $\alpha _{J_{i}}\sqrt {\mathbf {Var}\left [J_{i}\right ]}$. Since an objective function has to be minimized, an increase in variance is penalized by this reformulation. The reformulated robustified multi-objective dynamic optimization problem with deterministic constraints is formulated in Eq. (4).

4 $$  \begin{aligned} & \underset{\mathbf{u, x}, t_{\mathrm{f}}}{\text{min}} \quad \left\{\mathbf{E}\left[J_{1}\right]+\alpha_{J_{1}}\sqrt{\mathbf{Var}\left[J_{1}\right]}, \dots, \mathbf{E}\left[J_{n_{J}}\right]+\alpha_{J_{n_{J}}}\sqrt{\mathbf{Var}\left[J_{n_{J}}\right]}\right\} \\ \text{s.t.} & \left\{ \begin{array}{lll} \dot{\mathbf{x}} &=& \mathbf{f}(\mathbf{x},\mathbf{u},\boldsymbol{\theta},t) \\ \mathbf{x}(0) &=& \mathbf{x}_{0} \\ 0&\geq&\mathbf{E}\left[c_{\text{prob},i}\right]+\alpha_{c_{\text{prob},i}} \sqrt{\mathbf{Var}\left[c_{\text{prob},i}\right]}\\ & & i = 1, \hdots, n_{c_{\text{prob}}} \end{array} \right. \\ \end{aligned}  $$

In practice, not all constraints have to be replaced by probabilistic constraints (e.g., bounds on the controls and states) and constraints of the form **0**≥**c**(**x**,**u**,***θ***,*t*) can still be present in the optimization problem formulation.

### Approximation techniques for uncertainty propagation

**Fig. 1:**
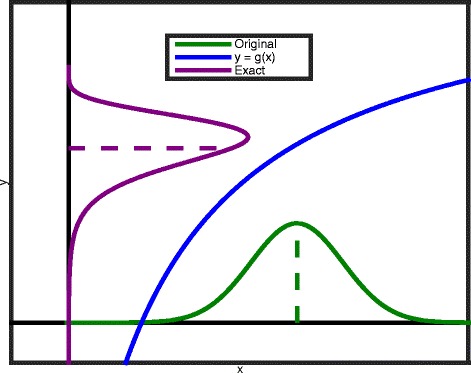
Principle of uncertainty propagation. Uncertainty propagation of *x* towards *y* via nonlinear transformation *g*(*x*) in an exact way

In this paper, the parametric uncertainty is propagated to the states, constraints and objectives of interest. This can be illustrated with an example shown in Fig. 1. Consider the simple nonlinear model *y*=*g*(*x*) (blue curve), with the parameter x that is uncertain with a known parametric uncertainty distribution (green curve). The principle of uncertainty propagation will propagate the parametric uncertainty distribution (green curve) through the nonlinear model (blue curve) in order to obtain the uncertainty distribution of the output *y* (purple curve).

The parametric uncertainty can be propagated via a numerical integration over the parameter distribution [21]. However, this integration is typically computationally expensive for realistic models and more efficient approximative uncertainty propagation techniques exist.

An alternative to this numerical integration is the use of Monte Carlo simulations in optimization. A large number of *N* realizations is drawn from the assumed parametric uncertainty distribution with variance-covariance matrix ***Σ*** and the empirical confidence regions can be determined by using the appropriate quantiles. However, this is in practice computationally extremely expensive. Due to the large amount of simulations, no computationally tractable procedure for gradient based optimization schemes is available. In addition there is no clear rule on how many noise realizations have to be taken in order to obtain an accurate estimate [10]. For these reasons, Monte Carlo simulations are not pursued in the dynamic optimization procedure.

Approaches that exploit the availability of measurements as described in [11, 22, 23] are also used in the field of robust optimization. However, these are not considered in this paper, since in an industrial setting intracellular measurements are typically not available on a routine basis.

The first type of the employed techniques is a so-called *linearization approach*, which is based on first-order Taylor series approximations of the model functions with respect to the uncertainty [10, 11]. This approximation can be used if higher order terms can be neglected. This is the case when the uncertainty is small compared to the model curvature [15]. In this linearization approach a linear approximation of the variance-covariance matrix [11] of the states is made. On the other hand efficient sampling-based uncertainty propagation techniques exist as, e.g., using Hammersley sequences [24], the unscented transformation or *sigma points approach* [12] and the *polynomial chaos expansion approach* [19, 25].

In addtition to the *linearization approach* [11], two other techniques are considered: the *sigma points approach* [12] and the *polynomial chaos expansion approach* [19].

In practice, one is often interested in the violation of a path constraint (e.g., fluxes or concentrations that should not exceed their bounds for cell viability), a terminal constraint (e.g., a minimum amount of a specific metabolite to be produced) or the robustness of the objective (e.g., a minimum enzymatic cost), i.e., minimizing the uncertainty on the objective by taking into account the variance on the objective. The constraint or objective function to which the uncertainty is propagated, is denoted by *R*_*k*_(**x**,**u**,***θ***,*t*) in the following. The three techniques consist of propagating the parametric uncertainty by approximating the expected value **E**[*R*_*k*_] and variance **V** **a** **r**[*R*_*k*_] of *R*_*k*_(**x**,**u**,***θ***,*t*). The approximated expected value and variance are denoted by $\bar {R}_{k,\text {LIN}}$, $\bar {R}_{k,\text {SP}}$, $\bar {R}_{k,\text {PCE}}$ and *P*_LIN_,*P*_SP_, *P*_PCE_, respectively.

**Fig. 2:**
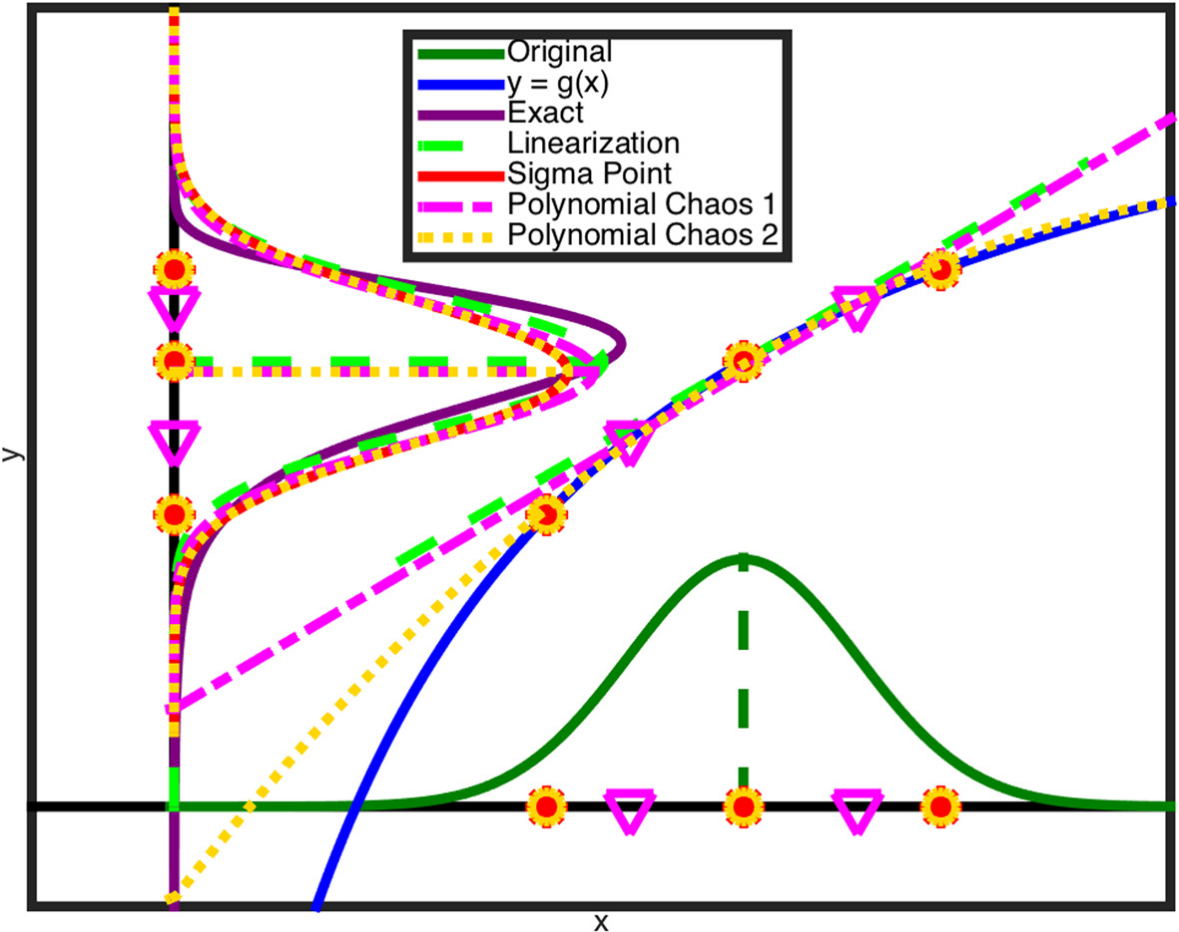
Approximation techniques for uncertainty propagation. Overview of the linearization, sigma points and polynomial chaos expansion methods for uncertainty propagation of *x* towards *y*=*g*(*x*)

An overview of these techniques with the robustified multi-objective dynamic optimization problem formulation is provided in Table 2. A graphical representation of the approximation techniques for uncertainty propagation is shown in Fig. 2. A more detailed review of these techniques is presented in Additional file 1. Note that these approaches are also applicable to uncertainty present in the model right hand side. However, in this work only the application to parametric uncertainty propagation is considered.

**Table 2:** Overview of the approximation techniques for uncertainty propagation with *R*_*k*_ the variable to which the parametric uncertainty is propagated

	Linearization	Sigma points	Polynomial chaos
Rationale	Linearization of state equations around ${\bar {\boldsymbol {\theta }}}$	Approximate distribution by a fixed number of	Approximate response of the model (at sampling
		parameters (sigma points)	points) as a *p*th order polynomial function of ***θ***
Uncertainty distribution	Normal	Any symmetric, unimodal distribution	Any
Equations	State equations + Sensitivity equations:	State equations for SPs:	State equations for sampling points:
	$\left \{\begin {array}{lll} \dot {\mathbf {S}}_{\mathrm {{LIN}}}(t) &=& \frac {\partial \mathbf {f}(\mathbf {x},\mathbf {u},\boldsymbol {\theta }_{\text {nom}},t)}{\partial \mathbf {x}} \mathbf {S_{\mathrm {{LIN}}}} + \frac {\partial \mathbf {f}(\mathbf {x},\mathbf {u},\boldsymbol {\theta }_{\text {nom}},t)}{\partial \boldsymbol {\theta }},\\ \mathbf {S_{\mathrm {{LIN}}}}(0) &=& \mathbf {0} \end {array} \right. $	$\dot {\mathbf {x}}_{i}=\mathbf {f}(\mathbf {x_{i}},\mathbf {u}, \mathbf {\boldsymbol {\pi }_{i}},t)$ with *i*=0,…,2*n*_*θ*_	$\dot {\mathbf {x}}_{i} = \mathbf {f}(\mathbf {x_{i}},\mathbf {u}, \mathbf {\boldsymbol {\pi }_{i}},t)$ with *i*=0,…,*n*_*s*_−1
Total *n*_*states*_	(*n*_*θ*_+1)*n*_*x*_	(2*n*_*θ*_+1)*n*_*x*_	$\frac {(n_{\theta }+p)!}{n_{\theta }!p!}n_{x}$
Sampling points	–	Sigma points: 2*n*_*θ*_+1	Collocation points: $\frac {(n_{\theta }+p)!}{n_{\theta }!p!}$
Expected value of *R*_*k*_	*R*_*k*_	$\frac {1}{n_{\theta }+\kappa }\left (\kappa R_{k}(\boldsymbol {\pi }_{0}) + \frac 12 \sum ^{2n_{\theta }}_{i=1}R_{k}(\boldsymbol {\pi }_{i}) \right)$	$a_{R_{k},0}^{(p)}$
Variance on *R*_*k*_	$\mathbf {P_{\mathrm {{R_{k}R_{k},LIN}}}} = \frac {\partial R_{k}}{\partial \mathbf {x}}\mathbf {P_{\mathrm {{LIN}}}}\left (\frac {\partial R_{k}}{\partial \mathbf {x}}\right)^{\top }$	$\mathbf {P_{\mathrm {{R_{k}R_{k},SP}}}} = \frac {1}{n_{\theta }+\kappa }\left (\kappa (R_{k}(\boldsymbol {\pi }_{0}) -\bar {R_{k}})(R_{k}(\boldsymbol {\pi }_{0})-\bar {R_{k}})^{\top } \right)$	$$ {\mathbf{P}}_{{\mathrm{R}}_{\mathrm{k}}{\mathrm{R}}_{\mathrm{k}},PCE}^{(p)}=\sum_{j=1}^{L-1}{\left({a}_{R_k,j}^{(p)}\right)}^2\mathbf{E}\left[{\varPhi}_j^2\left(\theta \right)\right] $$
		$+\frac {1}{n_{\theta }+\kappa }\left (\frac 12 \sum ^{2n_{\theta }}_{i=1}(R_{k}(\boldsymbol {\pi }_{i}) -\bar {R_{k}})(R_{k}(\boldsymbol {\pi }_{i}) -\bar {R_{k}})^{\top } \right)$	
	with: *P*_LIN_=*S*_LIN_(*t*)***Σ*** *S*_LIN_(*t*)^⊤^	with: $\left \{\begin {array}{lll} \mathbf {\boldsymbol {\pi }_{0}} &=& \boldsymbol {\theta }_{\text {nom}} \;, \\ \mathbf {\boldsymbol {\pi }_{i}} &=& \boldsymbol {\theta }_{\text {nom}}+\sqrt {(n_{\mathrm {\theta }}+\kappa){\boldsymbol {\Sigma }}}_{i} \;\text {with}\; i=1, \hdots, n_{\mathrm {\theta }} \;, \\ \mathbf {\boldsymbol {\pi }_{i}} &=& \boldsymbol {\theta }_{\text {nom}}-\sqrt {(n_{\mathrm {\theta }}+\kappa)\boldsymbol {\Sigma }}_{i-n_{\mathrm {\theta }}} \;\text {with}\; i=n_{\mathrm {\theta }}+1, \hdots, 2n_{\mathrm {\theta }} \;. \\ \kappa &=& 3-n_{\theta } \; \end {array}\right.$	with: **a**=(***Λ*** ***Λ*** ^⊤^)^−1^ ***Λ*** *R*_k,s_
Optimization problem	$\underset {\mathbf {u, x}, t_{\mathrm {f}}}{\text {min}} \quad \{J_{1}, \dots, J_{n_{J}}\}$	$\underset {\mathbf {u, x}, t_{\mathrm {f}}}{\text {min}} \quad \{J_{1}, \dots, J_{n_{J}}\}$	$\underset {\mathbf {u, x}, t_{\mathrm {f}}}{\text {min}} \quad \{J_{1}, \dots, J_{n_{J}}\}$
	$\bar {J_{i}}_{\text {LIN}}+ \alpha _{J_{i}} \sqrt {\mathbf {P_{\mathrm {{J_{i}J_{i},SP}}}}}$	$\bar {J_{i}}_{\text {SP}}+ \alpha _{J_{i}} \sqrt {\mathbf {P_{\mathrm {{J_{i}J_{i},SP}}} }}$	$\bar {J_{i}}_{\text {PCE}}^{(p)}+ \alpha _{J_{i}} \sqrt {\mathbf {P_{\mathrm {{J_{i}J_{i},PCE}}}}^{(p)}}$
	s.t. $\left \{ \begin {array}{lll} \dot {\mathbf {x}} &=& \mathbf {f}(\mathbf {x},\mathbf {u},\boldsymbol {\theta },t) \\ \dot {\mathbf {S}}_{\mathrm {{LIN}}}(t) &=& \frac {\partial \mathbf {f}(\mathbf {x},\mathbf {u},\boldsymbol {\theta }_{\text {nom}},t)}{\partial \mathbf {x}} \mathbf {S_{\mathrm {{LIN}}}} + \frac {\partial \mathbf {f}(\mathbf {x},\mathbf {u},\boldsymbol {\theta }_{\text {nom}},t)}{\partial \boldsymbol {\theta }},\\ \mathbf {S_{\text {LIN}}}(0) &=& \mathbf {0} \\ \mathbf {x}(0)&=&\mathbf {x}_{0} \\ 0 &\geq & \bar {c}_{\text {prob},i, \text {LIN}} \\ &&+ \alpha _{c_{\text {prob},i}} \sqrt {\mathbf {P_{\mathrm {c_{\text {prob},i},c_{\text {prob},i}, LIN}}}} \end {array} \right.$	s.t. $\left \{ \begin {array}{lll} \dot {\mathbf {x}}_{i}&=&\mathbf {f}(\mathbf {x_{i}},\mathbf {u}, \mathbf {\boldsymbol {\pi }_{i}},t) \text {with}\; i = 0, \hdots, 2n_{\theta } \\ \mathbf {x}(0)&=&\mathbf {x}_{0} \\ 0 &\geq & \bar {c}_{\text {prob},i, \text {SP}} \\ &&+ \alpha _{c_{\text {prob},i}} \sqrt {\mathbf {P_{\mathrm {c_{\text {prob},i},c_{\text {prob},i},SP}}}} \\ \end {array} \right.$	s.t. $$ \left\{\begin{array}{lll}{\dot{\mathbf{x}}}_i& =& \mathbf{f}\left({\mathbf{x}}_i,\mathbf{u},{\pi}_i,t\right)\\ &&\mathrm{with} i=0,\dots, {n}_s-1\\ {}\mathbf{x}(0)& =& {\mathbf{x}}_0\\ {}{{\mathbf{R}}_{\mathbf{k},\mathbf{s}}}^{(p)}& =& {\left({\varLambda}^{(p)}\right)}^{\top }{{\mathbf{a}}_{{\mathbf{R}}_{\mathbf{k}}}}^{(p)}\\ {}\mathrm{with} k=1,\dots, {n}_R\\ {}0& \ge & {\bar{c}}_{\mathrm{prob},i,\mathrm{P}\mathrm{C}\mathrm{E}}^{(p)}\\ {}+{\alpha}_{c_{\mathrm{prob},i}}\sqrt{{{\mathbf{P}}_{{\mathbf{c}}_{\mathbf{prob},\mathbf{i}},{\mathbf{c}}_{\mathbf{prob},\mathbf{i}},\boldsymbol{P}\boldsymbol{C}\boldsymbol{E}}}^{(p)}}\\ {}\mathrm{with} i=1,\dots, {n}_{c_{\mathrm{prob}}}\end{array}\right. $$

**Note:** The approximation techniques for uncertainty propagation can also be used to quantify the effect of parametric uncertainty on model predictions. If the controls are fixed, then an additional benefit could be that the uncertainty on the states can be displayed. This enhances the insight in whereto the system can evolve with an a priori known or assumed parametric uncertainty. In fact, in literature it has been pointed out that the polynomial chaos expansion could be considered as an alternative to Monte Carlo simulations, but with less computational cost [25].

### Multi-objective optimization methods

In practice, multiple objectives, which are very often conflicting with each other, have to be considered simultaneously, e.g., minimizing the enzyme consumption while maximizing the production of a certain metabolite. Therefore, a single optimal solution will not exist, but a set of *trade-off solutions*, called the Pareto front, is obtained when solving a multi-objective problem [16].

Two categories of methods can be distinguished for the calculation of Pareto fronts: *scalarization* methods ([26–30]) that convert the multi-objective optimization problem into a series of single objective optimization problems by using scalar variables, and *vectorization* methods [16, 32–34] that start from a population of candidate solutions that gradually evolve to the Pareto front. Scalarization methods can take advantage of fast and efficient gradient based methods to find an optimum for the series of single objectives, while vectorization methods often use derivative-free optimization methods as evolutionary or stochastic optimization approaches. In this work the Normal Boundary Intersection (NBI) method [30, 31], i.e., a scalarization method, is used. For a more detailed description of the frame of multi-objective dynamic optimization and the NBI method see, e.g., [35] and Additional file 2.

### Implementation and software

The dynamic optimization problems in this work are solved using a direct approach, in which first the optimal control problem is discretized into a nonlinear optimization problem that can be solved afterwards with NLP solvers. It is chosen to discretize the problems using an orthogonal collocation discretization scheme. The rationale of orthogonal collocation is that the states and controls are fully discretized with respect to time in finite elements. Per finite element there are four *collocation points* of which the first one is fixed and the three other ones should obey the model equations and are seen as equality constraints (i.e., so-called *collocation constraints*). Between each finite element there is also a constraint that ensures continuity (i.e., so-called *continuity constraints*). As interpolation between the *collocation points* a cubic Lagrange polynomial is used, with four collocation points situated at the Radau roots on each interval. State bounds are easily added in this technique. The fact that orthogonal collocation has hardly any problem with stiff systems is advantageous in case of numerically unstable systems.

An inhouse developed software package, called Pomodoro, is used for the implementation of both case studies. The Pomodoro software contains a collection of algorithms and tools for dynamic optimization and is implemented in Python. Pomodoro uses CasADi [36] as a backbone for the dynamic optimization problem formulation. CasADi is a software package for rapid prototyping of large-scale optimization problems with automatic differentiation using a symbolic/numeric approach. For solving the NLP, an interior point algorithm, IPOPT [37], has been used. The Pomodoro software can be downloaded from https://perswww.kuleuven.be/~u0093798/software.php. For review purposes the work describing Pomodoro (Bhonsale SS, Telen D, Vercammen D, Vallerio M, Hufkens J, Nimmegeers P, Logist F, Van Impe J. Pomodoro - A novel toolkit for (multiobjective) dynamic optimization, model based control and estimation, submitted), can be found on http://www.student.kuleuven.be/~s0212066/pomodoro/. For more information on the optimization methods and implementation, the reader is referred to Additional file 2 and [35].

### Case studies

**Fig. 3:**
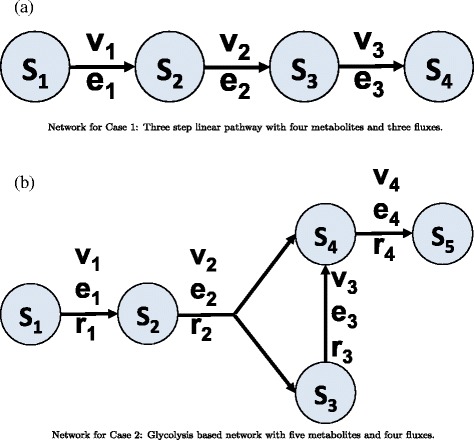
Networks for Case 1 and Case 2. Biological networks based on [7]: **a** Three step linear pathway with four metabolites and three fluxes and **b** Glycolysis based network with five metabolites and four fluxes

Two case studies are considered: *(a)* a three-step linear pathway with mass-action kinetics [7, 38] and *(b)* a glycolysis based network with 1 output [7, 39]. It should be noted that the models for the case study are partially taken from [7]. The networks for the two case studies are presented in Fig. 3.

#### Case 1: three-step linear pathway

The first case study is a three-step linear pathway producing one product *S*_4_ from a buffered substrate *S*_1_ [7, 38]. This pathway consists of three enzymatic reactions (with reaction fluxes **v**=[*v*_1_ *v*_2_ *v*_3_]^⊤^.) following mass-action kinetics, between 4 metabolites **S**=[*S*_1_ *S*_2_ *S*_3_ *S*_4_]^⊤^. Each reaction is catalyzed by a specific enzyme *e*_*i*_. The first metabolite *S*_1_ is the substrate, intermediate metabolites are *S*_2_ and *S*_3_, while *S*_4_ is the product, produced by this three step linear pathway. The substrate is considered to be buffered, which means that the substrate concentration remains constant.

The model contains four differential states and an additional state *x*_extra_ for the objective function corresponding to the integral of the intermediate accumulation. The model together with its constraints is presented in Eq. (5). The first constraint is to ensure that a minimum amount of product is obtained at *t*_f_. The second constraint expresses that the sum of all enzyme concentrations cannot exceed the total enzymatic concentration *E*_*T*_ of 1 mM. The enzyme concentrations **e**=[*e*_1_ *e*_2_ *e*_3_]^⊤^ are the controls in this case study.

5 $$ \begin{aligned}  &\left\{ \begin{array}{lll} {\frac{d\mathbf{S}}{dt}} &=& \mathbf{Nv} \\ {\frac{{dx}_{\text{extra}}}{dt}} &=& S_{2}+S_{3} \\ S_{4}(t_{\mathrm{f}}) &\geq& 0.90 \text{mM} \\ \sum_{i_{e}=1}^{n_{e}=3}e_{i}&\leq& E_{T} \\ \end{array} \right. \end{aligned}  $$

6 $$\begin{array}{*{20}l} & \text{with:} \\ & v_{1} = k_{1}\cdot S_{1}\cdot e_{1} \end{array} $$

7 $$\begin{array}{*{20}l} & v_{2} = k_{2}\cdot S_{2}\cdot e_{2} \end{array} $$

8 $$\begin{array}{*{20}l} & v_{3} = k_{3}\cdot S_{3}\cdot e_{3} \end{array} $$

9 $$\begin{array}{*{20}l} & \mathbf{N}= \left[ \begin{array}{ccc} 0 & 0 & 0 \\ 1 & -1 & 0 \\ 0 & 1 & -1\\ 0 & 0 & 1 \\ \end{array}\right] \end{array} $$

with $\mathbf {N}\in \mathbb {R}^{4\times 3}$ the stoichiometric matrix, containing the stoichiometric coefficients *N*_*ij*_ of metabolite *i* in the *j*-th reaction and *k*_*j*_, the maximum reaction rate of reaction *j*.

The three uncertain parameters are the three reaction rate constants *k*_1_, *k*_2_ and *k*_3_, for which the nominal value equals 1 (mMs) ^−1^. Since high concentrations of intermediate metabolites *S*_2_ and *S*_3_ can be harmful for cell viability, the intermediate accumulation is minimized for this case study.

10 $$\begin{array}{*{20}l}  J = x_{\text{extra}}(t_{\mathrm{f}}) = \int\limits_{t_{0}=0 s}^{t_{\mathrm{f}}}S_{2}+S_{3} \text{} dt \end{array} $$

#### Case 2: glycolysis based network with 1 output

The second case study is a glycolysis based network with the production of one product *S*_5_, starting from one substrate *S*_1_ from [7]. In this pathway four enzymatic reactions are taking place, each catalyzed by a specific enzyme. The fluxes are modeled with Michaelis-Menten kinetics. The intracellular metabolites in this network are *S*_2_, *S*_3_ and *S*_4_. It is assumed that the substrate *S*_1_ is buffered. This case study is particularly interesting due to the branch that is present. Such branches often occur in biological networks and the presented problem formulation can be modified/extended to many scenarios.

The expressions for this model are presented in Eq. (11) where **N** is the stoichiometric matrix, with **v**=[*v*_1_ *v*_2_ *v*_3_ *v*_4_]^⊤^ the flux vector, **S**=[*S*_1_ *S*_2_ *S*_3_ *S*_4_ *S*_5_]^⊤^ the vector containing the metabolite concentrations, **e**=[*e*_1_ *e*_2_ *e*_3_ *e*_4_]^⊤^ the enzyme concentration vector, the vector of manipulated variables **r**=[*r*_1_ *r*_2_ *r*_3_ *r*_4_]^⊤^ containing the expression rates, *k*_cat,*j*_ the maximum reaction rate for reaction *j*, dependent on the enzyme that is catalyzing the reaction *j* which is assumed to be the same for each reaction and therefore considered as the model parameter *k*_cat_ and *K*_*M*_ the Michaelis constant.

11 $$\begin{array}{*{20}l}  \left\{ \begin{array}{lll} \frac{d\mathbf{S}}{dt} &=& \mathbf{Nv} \\ \frac{d\mathbf{e}}{dt} &=& \mathbf{r}-\lambda\mathbf{e} \\ \frac{{dx}_{\text{extra}}}{dt} &=& e_{1}+e_{2}+e_{3}+e_{4} \\ S_{5}(t_{\mathrm{f}}) &\geq& 0.675 \text{ mM} \\ \sum_{i_{e}=1}^{n_{e}=4}e_{i}&\leq& E_{T} \\ r_{1}(t_{0})&=& 0.5 \\ r_{i_{r}}(t_{0})&=& 0, \sum_{i_{r}=1}^{n_{r}=4}r_{i_{r}}\leq 0.5\quad i_{r}=2,\dots, n_{r}=4 \end{array} \right. \end{array} $$

12 $$\begin{array}{*{20}l}  \text{with:}& \\ v_{1} &= \frac{k_{\text{cat}}\cdot S_{1}}{K_{M}+S_{1}}\cdot e_{1} \end{array} $$

13 $$\begin{array}{*{20}l} v_{2} &= \frac{k_{\text{cat}}\cdot S_{2}}{K_{M}+S_{2}}\cdot e_{2} \end{array} $$

14 $$\begin{array}{*{20}l} v_{3} &= \frac{k_{\text{cat}}\cdot S_{3}}{K_{M}+S_{3}}\cdot e_{3} \end{array} $$

15 $$\begin{array}{*{20}l} v_{4} &= \frac{k_{\text{cat}}\cdot S_{4}}{K_{M}+S_{4}}\cdot e_{4} \end{array} $$

16 $$\begin{array}{*{20}l} \mathbf{N}&= \left[ \begin{array}{cccc} 0 & 0 & 0 & 0 \\ 1 & -1 & 0 & 0 \\ 0 & 1 & -1 & 0 \\ 0 & 1 & 1 & -1 \\ 0 & 0 & 0 & 1 \\ \end{array}\right] \end{array} $$

The following values for the parameters are assumed [7]: *k*_cat_=1 s ^−1^, *K*_*M*_=1 mM and *λ*=0.5 s ^−1^.

Two objectives are considered for which the enzyme expression rates in **r** are optimized: the minimization of the time to reach a given steady state and the enzyme consumption (or enzymatic cost) as shown in Eqs. (17)-(18):

17 $$ \begin{aligned} J_{1} =& \; t_{\mathrm{f}} \end{aligned}  $$

18 $$ {\small{\begin{aligned} J_{2} =& \int\limits_{t_{0}=0 s}^{t_{\mathrm{f}}}\left(\sum\limits_{i_{e}=1}^{n_{e}=4}e_{i_{e}}dt\right). \end{aligned}}}  $$

## Results and discussion

This section discusses the obtained results in this work. In the first subsection the approach followed to obtain these results is clarified. Next, the results for the three-step linear pathway are presented. In the third subsection the results for the glycolysis inspired network are described.

### Approach

**Fig. 4:**
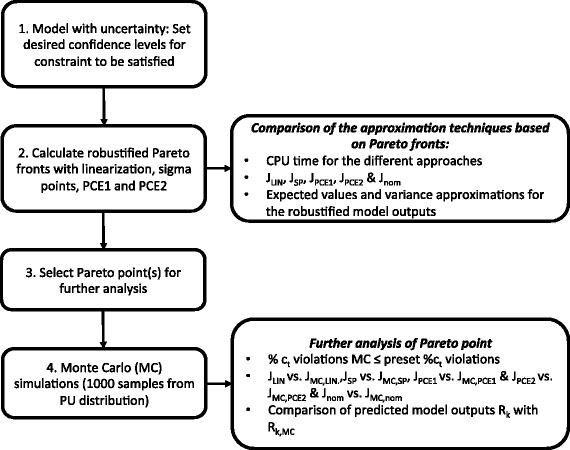
Illustration of the approach. Different steps in the approach followed in this work

The approach consists of the four steps in Fig. 4. This approach is formulated for the generic case of the multi-objective dynamic optimization of biological networks under uncertainty. First, the (multi-objective) dynamic optimization problem is solved for the nominal (non-robustified) case. Then, desired confidence levels for the robustified constraint are set (*Step 1*). Robustified terminal constraints of the form $c_{\text {min}} \leq \mathbf {E}\left [c(t_{\mathrm {f}})\right ] - \alpha _{c} \sqrt {\mathbf {Var}\left [c(t_{\mathrm {f}})\right ]}$ are considered. Table 1 presents the backoff parameter values that are used for the computation of the robustified controls together with the corresponding quantiles and preset confidence levels for a normal distribution of the constraint.

In *Step 2* robustified Pareto fronts are calculated with the linearization, sigma points and polynomial chaos expansion approaches (PCE1 and PCE2) to include parametric uncertainty in the multi-objective dynamic optimization problem. These robustified Pareto fronts are computed for the different backoff parameter values.

A first comparison of the approximation techniques for uncertainty propagation is based on the Pareto fronts: comparison of the CPU time for a single Pareto point, comparison of the objective function vectors **J**_LIN_, **J**_SP_, **J**_PCE1_ and **J**_PCE2_, calculated with the linearization, sigma points, PCE1 and PCE2 respectively. Also the expected values and variance approximations for the robustified model outputs with the approximation techniques for uncertainty propagation are compared.

Subsequently, a Pareto point is selected from the Pareto front for further analysis (*Step 3*). This analysis can be performed for any Pareto point or confidence level set (i.e., corresponding to different backoff parameter values *α*_*i*_), without a loss of generality. In this work, the considered point corresponds to one of the objectives, i.e., the minimization of the intermediate accumulation for Case 1 (single objective case study) and the minimization of the enzymatic cost for Case 2 (multi-objective case study).

In *Step 4*, Monte Carlo simulations are done for the considered Pareto point by sampling 1000 randomly generated parameter sets from the parametric uncertainty distribution. The robustified controls, determined with linearization, sigma points and polynomial chaos expansion approaches (PCE1 and PCE2), are fixed in the Monte Carlo simulations.

The further analysis of the Pareto point consists of assessing the robustness of the optimal control profiles obtained with the different approximation techniques for uncertainty propagation: i.e., *(i)* checking the reduction of constraint violations by applying a robustified control in comparison with applying a nominal (non-robustified) control profile, *(ii)* evaluating the backoff taken in objective function when uncertainty is taken into account and *(iii)* a comparison between the predicted expected value and variance for the robustified model output with the approximation techniques for uncertainty propagation and the calculated mean and variance with the Monte Carlo simulations. Furthermore, for the robustified single chance constraint it is investigated whether the preset confidence is reached for different backoff parameter values. This is done by checking whether the percentage of constraint violations in Monte Carlo simulations does not exceed the preset percentage of constraint violations. For instance, a confidence level of 0.95 is associated with a backoff parameter value of 1.65, meaning that in case the robustified control is applied, the percentage of constraint violations is not allowed to exceed 5 %. Alternatively, if the confidence level is not sufficient, the preset confidence can be increased by increasing the backoff parameter. An iterative procedure to determine the quantiles and backoff parameter can be followed as presented in [10].

Both case studies have been implemented in Pomodoro. The KKT tolerance is set to 10^−5^ and an orthogonal collocation discretization scheme is used for the dynamic optimization problems. Since the polynomial chaos expansion allows to take a priori information on the parametric uncertainty distribution directly into account via the orthogonal polynomials, two parametric uncertainty distributions have been studied: a priori normal and a priori uniform parametric uncertainty distribution with as mean the nominal parameter values and 20 % relative standard deviation in Case 1 and 10 % relative standard deviation in Case 2. For the normal parametric uncertainty distribution, Hermite polynomials are used, while for the uniform distribution another type is used. The reader is referred to Additional files 3 and 4.

### Case 1: three step linear pathway

In this case study the terminal constraint expressing that the concentration of *S*_4_ at time *t*_f_ should exceed or equal 0.90 mM is robustified.

19 $$\begin{array}{*{20}l}  0.90\text{mM} &\leq \mathbf{E}\left[S_{4}(t_{\mathrm{f}})\right] - \alpha_{S_{4}} \sqrt{\mathbf{Var}\left[S_{4}(t_{\mathrm{f}})\right]} \end{array} $$

Since this constraint only looks at one bound that has to be exceeded, the 95 % confidence region should be covered when a backoff parameter of $\alpha _{S_{4}}=1.65$ is chosen. The intermediate accumulation objective function is not robustified for this case study (i.e., $\alpha _{J_{2}}$). The three uncertain parameters are *k*_1_, *k*_2_ and *k*_3_.

In this section, the single objective optimization (i.e., the minimization) of the intermediate accumulation is considered. Intermediate accumulation can be harmful for the cell and should therefore be minimized. It is assumed that the final time is fixed at 10 seconds. First the computational aspects are discussed. Subsequently a physical/biological interpretation of the results is given. To conclude Case 1 the approximation techniques for uncertainty propagation are compared based on the results from the single objective optimization and Monte Carlo simulations for a normal and uniform parametric uncertainty distribution.

#### Computational aspects

A first aspect is the computational cost of including uncertainty in dynamic optimization. The number of required states and variables, together with the CPU time for the different approximation techniques for uncertainty propagation are presented in Table 3. The CPU times are presented for the largest backoff parameter used in this work, i.e., $\alpha _{S_{4}}=1.96$, to give an upper bound on the computation times that are required. The results in Table 3 confirm that taking uncertainty into account, leads to an increased computational time. An inherent property of the considered uncertainty propagation techniques, is the increase in number of states when the number of uncertain parameters increases. The increase in computational time for this case study is thus related to the increase in the size of the optimization problem. From this it is clear that the linearization and PCE1 approaches have a similar computation time and are the fastest of the considered approximation techniques for uncertainty propagation, followed by the sigma points approach and PCE2 approach.

**Table 3:** Case 1 - Overview of the number of states, CPU time, expected values of the objective function, expected values of the terminal constraint and standard deviations for the different approximation techniques for uncertainty propagation when the enzymatic cost is minimized for *α*=1.96 for 3 uncertain parameters *k*_1_, *k*_2_ and *k*_3_

	Nominal	Linearization	Sigma points	PCE1	PCE2
States	5	20	29	17	41
CPU time [s]	0.156	1.332	4.507	2.087	7.780
**E**[*J*]	3.65	6.61	7.06	7.74	7.04
**E**[*S*_4_]	0.90	1.50	1.52	1.53	1.52
$\sqrt {\mathbf {Var}[\!S_{4}]}$	0	0.30	0.32	0.33	0.32

#### Physical/biological interpretation

**Fig. 5:**
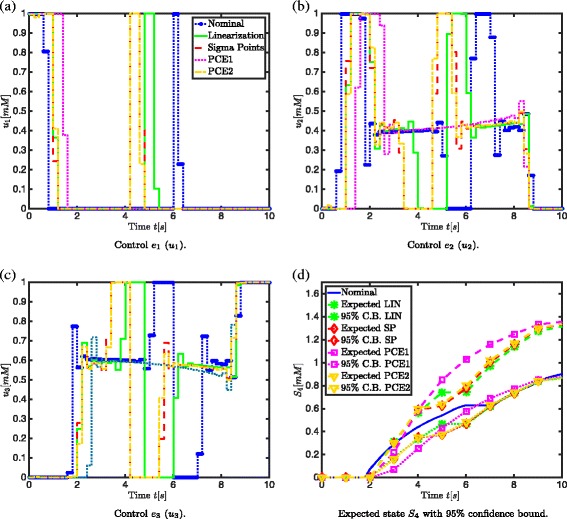
Results for Case 1. Comparison of the control profiles *e*_1_ (**a**), *e*_2_ (**b**) and *e*_3_ (**c**) calculated with linearization, sigma points approach, PCE1 and PCE2 for *α*=1.65 with the nominal control profile and (**d**) comparison of the expected state *S*_4_ and its 95 % confidence bound calculated with linearization, sigma points, PCE1 and PCE2 with the nominal case (*α*=1.65) in case of 3 uncertain parameters *k*_1_, *k*_2_ and *k*_3_

The enzyme concentration profiles are (from a computational point of view) seen as the optimal controls. The enzyme concentration profiles are illustrated in Fig. 5(a)-(c) for *α*=1.65. Different phases can be distinguished in the process (i.e., a sequential activation of the controls): *(i)* a first phase in which enzyme *e*_1_ is activated to produce *S*_2_ as fast as possible, *(ii)* a second phase in which both *S*_2_ and *S*_3_ are consumed and produced (by activation of *e*_2_ and *e*_3_) *(iii)* a third phase in which a novel activation of *e*_1_ takes place, followed by *(iv)* a phase of activity of *e*_2_ and *e*_3_ and a final phase in which the third enzyme is fully activated for the production of *S*_4_.

From the enzyme concentration profiles in Fig. 5(a)-(c), it is clear that, the robustified enzyme concentrations will increase earlier than the nominal enzyme concentrations. The sequential activation of the controls (i.e., the increasing and decreasing enzyme concentrations *e*_*i*_) will be early enough and sufficient in the robustified case to ensure that the robustified constraint is satisfied, i.e., a sufficient amount of *S*_4_ is produced. In Fig. 5(d) it is shown that for the PCE1 approach the minimum treshold of 0.90 *mM* is reached after 5.3 seconds, for PCE2 after 6.52, for the sigma points after 6.58 and for linearization after 6.7 seconds, while in the nominal case the minimum treshold of 0.90 *mM* is reached at the end time of 10 seconds. The enzyme concentrations, calculated with the approximative uncertainty propagation techniques, ensure more robustness towards satisfying the minimum end concentration of 0.90 *mM* for *S*_4_. However, including robustness towards satisfying the minimum end concentration leads to a higher intermediate accumulation as shown in Table 3. This is acceptable, as long as cell viability is not compromised. Comparing this with experimental results for amino-acid biosynthetic pathways in *Escherichia coli* [38, 40, 41] a sequential activation of the enzymes can be observed in the enzyme concentration profiles.

#### Comparison of the approximation techniques for uncertainty propagation

The number of constraint violations, expected value of *S*_4_ and the variance on *S*_4_ for the different approaches and $\alpha _{S_{4}}$ are shown in Tables 4 and 5 for a normal and uniform parametric uncertainty distribution, respectively. More extensive results are given in Additional file 3.

**Table 4:** Case 1 - Results Monte Carlo simulations (*N*=1000) in case of three normally distributed uncertain parameters *k*_1_, *k*_2_ and *k*_3_ for robustified terminal constraint with the number of constraint violations, mean terminal constraint values and variance on the terminal constraint

	Nominal case	Linearization	Sigma points	PCE 1	PCE 2
*α*=1.96					
$\bar {J}$	11.903	13.4600	13.600	13.829	13.583
*σ*_*J*_	0.5578	0.9737	1.0087	1.016	0.9981
$\bar {S_{4}}$	0.88734	1.4739	1.5242	1.5500	1.5316
$\sigma _{S_{4}}$	0.1733	0.2878	0.2970	0.2993	0.2993
*c*_*t*_ violations	509 (50.9 %)	30 (3.0 %)	21 (2.1 %)	20 (2.0 %)	20 (2.0 %)
*α*=1.65					
$\bar {J}$	11.903	13.051	13.127	13.331	13.139
*σ*_*J*_	0.5578	0.8624	0.8869	0.9049	0.8892
$\bar {S_{4}}$	0.8712	1.3357	1.3765	1.3910	1.3781
$\sigma _{S_{4}}$	0.1733	0.2614	0.2691	0.2683	0.2690
*c*_*t*_ violations	509 (50.9 %)	51 (5.1 %)	44 (4.4 %)	39 (3.9 %)	43 (4.3 %)

**Table 5:** Case 1 - Results Monte Carlo simulations (*N*=1000) in case of three uniformly distributed uncertain parameters *k*_1_,*k*_2_ and *k*_3_ for robustified terminal constraint with the number of constraint violations, mean values and variances on the objective function and terminal constraint, respectively

	Nominal case	Linearization	Sigma points	PCE1	PCE2	PCE2 Uniform
*α*=1.96						
$\bar {J}$	11.913	13.477	13.618	13.847	13.601	13.591
*σ*_*J*_	0.5470	0.9601	0.9942	1.0000	0.9835	0.9790
$\bar {c_{t}}$	0.88917	1.4768	1.5273	1.5533	1.5347	1.5261
$\sigma _{c_{t}}$	0.1818	0.3015	0.3110	0.3132	0.3137	0.3118
*c*_*t*_ violation	516 (51.6 %)	4 (0.4 %)	1 (0.1 %)	0 (0.0 %)	1 (0.1 %)	2 (0.2 %)
*α*=1.65						
$\bar {J}$	11.913	13.066	13.143	13.347	13.155	13.330
*σ*_*J*_	0.5470	0.8489	0.8730	0.6813	0.8750	0.8881
$\bar {c_{t}}$	0.88917	1.3384	1.3792	1.3940	1.3809	1.3660
$\sigma _{c_{t}}$	0.1818	0.2740	0.2820	0.2808	0.2819	0.2754
*c*_*t*_ violation	516 (51.6 %)	35 (3.5 %)	22 (2.2 %)	11 (1.1 %)	22 (2.2 %)	22 (2.2 %)

**Expected value and 95 % confidence bound** First the expected value and 95 % confidence bound of *S*_4_ (based on $\alpha _{S_{4}}=1.65$) are compared. This is done in Fig. 5(d) and it can be seen that the expected state and 95 % confidence bounds for *S*_4_ are very similar when computed with the linearization, sigma points and PCE2 approaches. The expected value for the PCE1 approach differs slightly from the others: initially it is taking more distance from the nominal profile, indicating that this approach is more conservative than the other approaches and will lead to less constraint violations. In general, linear approximation techniques (as the PCE1 approach) tend to be more conservative, but it cannot be predicted upfront whether this is in a positive sense or not.

**Constraint violations** In order to investigate the performance of the different approximation techniques for uncertainty propagation with respect to the constraint violations, a Monte Carlo simulation with 1000 realizations (i.e., randomly generated parameter samples from the parametric uncertainty distribution) has been performed for the four approaches and is compared with the nominal case.

From these simulations, it is observed in case of a normal parametric uncertainty distribution that all four methods reduce the amount of constraint violations significantly: from 50.9 % in the nominal case to even 2.0 % for PCE1 and PCE2, when a backoff parameter value of *α*=1.96 is chosen. The same holds for a uniform parametric uncertainty distribution.

In practice, the most interesting backoff parameter values are 1.65 and 1.96, corresponding to 5 % and 2.5 % violations in case of a normal distribution. From the results in Tables 4 and 5 it is clear that the PCE1 method scores the best with respect to constraint violations, followed by PCE2 and sigma points. The performance of the sigma points method and the second order polynomial chaos expansion are, as shown throughout this case study, very similar.

**Parametric uncertainty distribution** If an uncertainty distribution is assumed for the constraint and the back-off parameters are chosen in accordance with the quantiles (which is the case for the normal parametric uncertainty distribution), the level of constraint violations should correspond exactly with the confidence level. A too low degree of violations is also not wished, since the uncertainty is not propagated correctly in that case.

Furthermore, the expected values and variances of *S*_4_ for a backoff parameter of *α*=1.96 from Table 3 which are predicted with the approximation techniques, are very close to the empirically calculated expected values and variances by Monte Carlo simulation in Tables 4 and 5. For the sigma points, PCE1 and PCE2 approaches these predicted expected values and variances are an accurate estimation of the ones obtained by Monte Carlo simulation. For the linearization approach, this is not the case. However, the expected value of the intermediate accumulation objective function is not accurately predicted. This objective function is not robustified in this case study, as the variance on the objective function is not taken into account. Therefore, the predictions of the expected value of the objective functions are not accurate, when compared with a Monte Carlo setting.

There is less backoff from the objective function when a uniform parametric uncertainty distribution is assumed, also the variance is reduced for the second order polynomial chaos expansion with respect to the assumption of a normal parametric uncertainty distribution. The percentage of constraint violations on the other hand is slightly higher when a uniform distribution is considered. However, for this case study, the difference in performance between a uniform parametric uncertainty distribution and a normal parametric uncertainty distribution for the polynomial chaos expansion, is small. Therefore, it should be stressed that including the additional information on the parametric uncertainty distribution can be useful. However, gathering information on the parametric uncertainty distribution is quite intensive and does not lead to a drastic improvement in performance for this case study.

### Case 2: glycolysis based network with 1 output

The terminal constraint and enzymatic cost objective function are robustified in this case study as shown in following equations. This is done in order to reduce the variance on the objective function. In contrast to Case 1, this should allow to have a better prediction of the expected value and variance on the objective function.

20 $$\begin{array}{*{20}l} 0.675\text{mM} &\leq \mathbf{E}\left[S_{5}(t_{\mathrm{f}})\right] - \alpha_{S_{5}} \sqrt{\mathbf{Var}\left[S_{5}(t_{\mathrm{f}})\right]} \end{array} $$

21 $$\begin{array}{*{20}l} J_{2} &= \mathbf{E}\left[x_{\text{extra}}(t_{\mathrm{f}})\right] + \alpha_{x_{\text{extra}}} \mathbf{Var}\left[x_{\text{extra}}(t_{\mathrm{f}}))\right] \end{array} $$

For simplicity, the backoff parameters $\alpha _{S_{5}}$ and $\alpha _{x_{\text {extra}}}$ are assumed to be the same and are called *α* in the remainder of the text. The objective function is robustified by adding the term $\alpha _{x_{\text {extra}}} \mathbf {Var}\left [x_{\text {extra}}(t_{\mathrm {f}}))\right ]$, since the objective function has to be minimized and an increase in variance is penalized.

In this case study three parameters (*k*_cat_, *K*_*M*_ and *λ*) are considered uncertain. First, the multi-objective optimization results are discussed, followed by a more in depth analysis of the minimization of the enzymatic cost. Note that this in depth analysis can be performed for any Pareto point. Subsequently the computational aspects, a physical/biological interpretation of the results and comparison of the approaches are presented for this case study, based on the dynamic optimization results and Monte Carlo simulations for a normal and uniform parametric uncertainty distribution. For more extensive results, the reader is referred to Additional file 4.

#### Multi-objective optimization results

The multi-objective optimization problem consists of minimizing the time needed to reach at least 0.675 mM of product *S*_5_ and minimizing the enzymatic cost, i.e., the total enzyme consumption over the whole time span.

In the robustified problem formulation, both the objective function for the enzymatic cost and the terminal constraint are robustified with backoff parameter *α*. It is assumed that the final time *t*_f_ cannot exceed 30 seconds. The two objectives, final time and enzymatic cost, are clearly conflicting: reducing the time needed to reach a level of 0.675 mM of *S*_5_ leads to an increase in the enzymatic cost and vice versa.

**Fig. 6:**
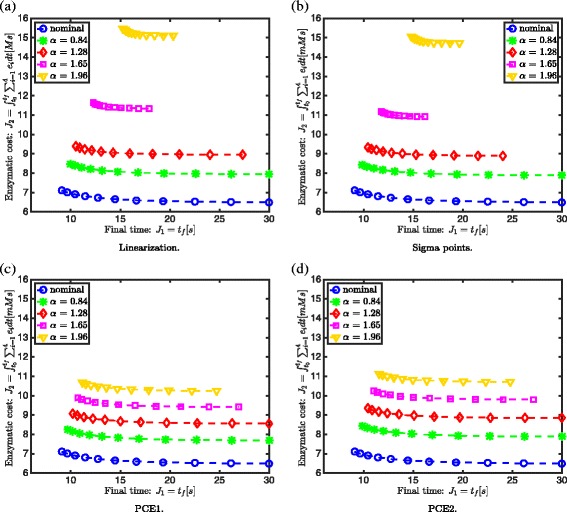
Receeding Pareto fronts Case 2. Receeding Pareto fronts with increasing backoff parameter *α* for linearization (**a**), sigma points (**b**), first (**c**) and second order polynomial chaos expansion (**d**) approach in case of 3 uncertain parameters *K*_*M*_, *λ* and *k*_cat_

Receeding Pareto fronts from the nominal optimal solution with increasing backoff parameter values can be observed in Fig. 6: both anchor points shift away from the nominal optimal solution. This is the price in performance (i.e., minimum time and enzymatic cost) that has to be paid to ensure a minimum concentration of 0.675 mM for *S*_5_. However, it is also observed that the Pareto fronts change shape and range, when the backoff parameter increases. This is related to the feasibility of the Pareto points.

A comparison of the different approximation techniques for uncertainty propagation based on the Pareto fronts is presented in Additional file 4. From this comparison it is firstly seen that the linearization and sigma points approach take more backoff than the polynomial chaos approaches. Secondly, when the backoff parameters decrease, it is observed that there is a similarity between the Pareto fronts for the PCE2 and sigma points approach. This can be explained from the variance that is taken less into account when the backoff parameter value decreases. Since the difference between the PCE2 and sigma points approach lies in the variance calculation, this explains the increasing difference in Pareto fronts with an increasing backoff parameter value. The expected value calculation is the same for the sigma points and PCE2 approach in case of a normal parametric uncertainty distribution. For this case study, the sigma points are a subset of the PCE2 sampling points as shown in Additional file 4.

#### Computational aspects

For the discussion of the computational aspects, the single objective optimization of the enzymatic cost is considered. It is assumed that the final time is fixed at 30 seconds.

In Table 6 an overview is presented of the number of states, the CPU time, objective function values, terminal constraint and their expected values and standard deviations for the different approximation techniques for uncertainty propagation for 3 uncertain parameters *K*_*M*_, *λ* and *k*_cat_. This is done for the largest backoff parameter value *α*=1.96 for the same reasons as mentioned in the first case study.

**Table 6:** Case 2 - Overview of the number of states, CPU time, objective function values, terminal constraint values and their expected values and standard deviations for the different approximation techniques for uncertainty propagation when the enzymatic cost is minimized for *α*=1.96 for 3 uncertain parameters (*K*_*m*_, *λ* and *k*_cat_)

	Nominal	Linearization	Sigma points	PCE1	PCE2
States	9	36	63	36	90
CPU time [s]	0.547	117.474	25.65	4.574	25.847
**E**[*J*]	6.500	12.945	8.338	8.799	9.168
$\sqrt {\mathbf {Var}[J]}$	0	1.113	0.697	0.739	0.791
*c*_*t*_	0.675	0.675	0.675	0.675	0.675
**E**[*c*_*t*_]	0.675	1.000	1.067	1.163	1.210
$\sqrt {\mathbf {Var}[c_{t}]}$	0	0.166	0.200	0.249	0.273

In this case study the linearization approach is computationally the most expensive. While in Case 1, the increase in computational time is related to the increase in the size of the optimization problem, this cannot be the explanation for why the linearization approach takes the most CPU time. One explanation for the long CPU time of the linearization approach, can be the nonlinearity of the model in Case 2 and solving the sensitivity equations. The interconnection of the states in the sensitivity equations, makes the linearization approach computationally more challenging.

#### Physical/biological interpretation

**Fig. 7:**
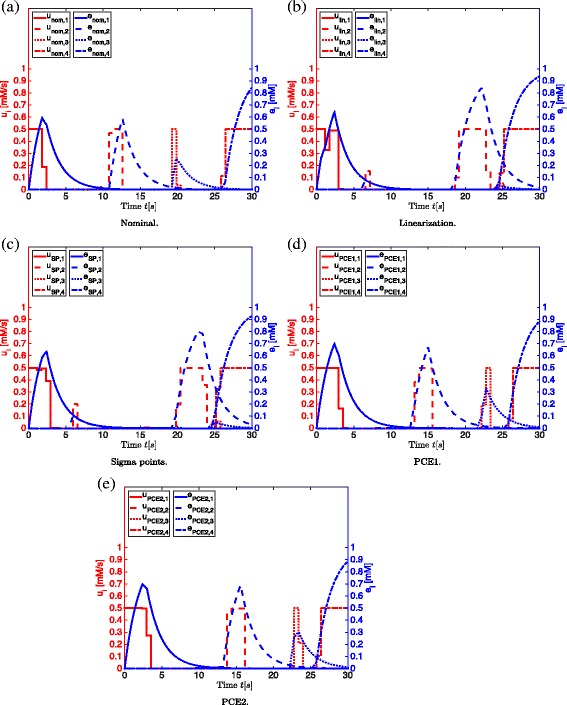
Enzyme expression rates and enzyme concentration profiles Case 2. Enzyme expression rates together with the enzyme concentration profiles following from the minimization of the enzymatic cost for the nominal case (**a**) and with the different approximation techniques for uncertainty propagation: linearization (**b**), sigma points (**c**), PCE1 (**d**) and PCE2 (**e**) (*α*=1.65)

**Fig. 8:**
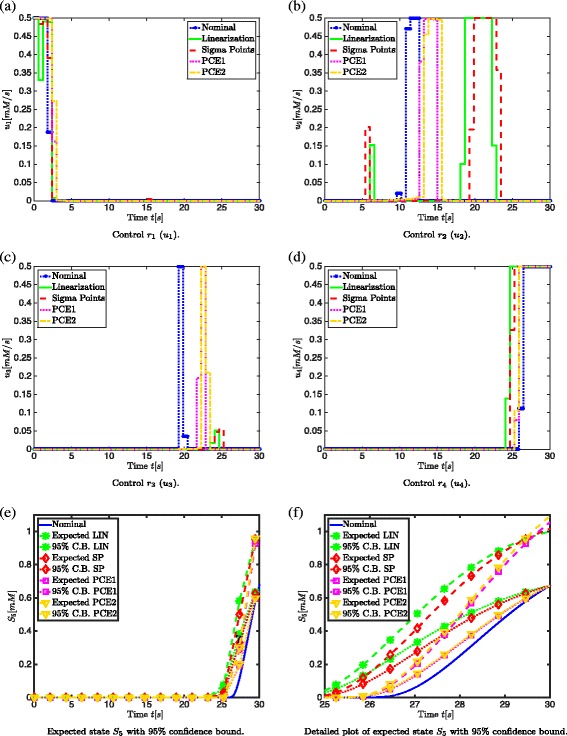
Results for minimization of enzymatic cost in Case 2. Comparison of the control profiles *r*_1_ (**a**), *r*_2_ (**b**), *r*_3_ (**c**) and *r*_4_ (**d**) calculated with linearization, sigma points approach, PCE1 and PCE2 for *α*=1.65 with the nominal control profile and comparison of expected state *S*_5_ and its 95 % confidence bound calculated with linearization, sigma points, PCE1 and PCE2 with the nominal case (*α*=1.65) ((**e**) and (**f**)) in case of 3 uncertain parameters *K*_*M*_, *λ* and *k*_cat_

In Fig. 7 the enzyme expression rates are shown together with the corresponding enzyme concentration profiles, which are computed by minimizing the enzymatic cost for the nominal case (a) and with the different approximation techniques for uncertainty propagation: linearization (b), sigma points (c), PCE1 (d) and PCE2 (e) (*α*=1.65). It is observed that the expression rate profiles show an on/off behavior that leads to the sequential activation of the different enzymes in the network. The physical interpretation of this on/off behavior of the enzyme expression rates is that the previous enzyme first has to be degraded, before the other enzyme can be synthesized. This makes sense from a biological point of view, since the cells only have a limited amount of proteins available. This also corresponds to the satisfaction of the constraint on the total enzymatic content. When minimizing the end time, there is a high accumulation of metabolites. This accumulation can affect cell viability negatively. The optimal control profiles in Fig. 8(a)-(d) clearly show a switching pattern, corresponding to the sequential activation of the pathways. According to [42] there is a mechanism leading to more pronounced transcriptional control of costly enzymes which can be explained by the trade-off between enzymatic cost minimization and time. Similarly to the Case 1, it can be seen in Fig. 8(e)-(f) that the minimum threshold of 0.675 *mM* for *S*_5_ is reached sooner when applying the approximation techniques for uncertainty propagation. However, this robustness with respect to the terminal constraint on *S*_5_ comes together with an increase in enzymatic cost as shown in Table 6. To avoid a too high enzymatic cost that is harmful for cell viability, an upper bound on the enzymatic cost can be introduced and robustified.

In Fig. 7(b) and (c) some remarkable observations are made for the linearization and sigma points approaches. After approximately 6 seconds a small expression rate *u*_2_ is observed, leading to a short activation of *e*_2_, producing intermediates *S*_3_ and *S*_4_. Furthermore after approximately 18 seconds in the linearization approach and 20 seconds in the sigma points approach, *e*_2_ is expressed for a longer time. The accumulated *S*_2_ is intensively consumed in this period for the production of intermediates *S*_3_ and *S*_4_. Eventually this will lead to a very low concentration of *S*_2_ in comparison with the other approaches. Furthermore, a very small expression rate *u*_3_ is observed in Fig. 7(b) and (c) for the optimization with the linearization and sigma points approaches, leading to a weak activation of *e*_3_. This means that the branch in the glycolysis inspired network involving the conversion of *S*_3_ to *S*_4_ is practically inactive, leading to accumulation of *S*_3_ for the linearization and sigma points approaches. This behavior is not observed in the profiles obtained with the other techniques and explains the higher objective function values for enzymatic cost in case of the linearization and sigma points approaches.

#### Comparison of the approximation techniques for uncertainty propagation

The performance of the different approximation techniques for uncertainty propagation with respect to the constraint violations is investigated by performing a Monte Carlo simulation with 1000 noise realizations from a normal distribution. The number of constraint violations on *S*_5_, mean values, and the variances for the objective function and terminal constraint, respectively are shown in Table 7 for the different approaches and backoff parameter values *α*_*i*_. More extensive results are presented in Additional file 4.

**Table 7:** Case 2 - Results Monte Carlo simulations (*N*=1000) in case of three normally distributed uncertain parameters (*K*_*m*_, *λ* and *k*_cat_) for robustified terminal constraint and objective function with the number of constraint violations, mean values and variances on the objective function and terminal constraint, respectively

	Nominal case	Linearization	Sigma points	PCE1	PCE2
*α*=1.96					
$\bar {J}$	6.5268	12.996	12.549	8.7702	9.1358
*σ*_*J*_	0.57543	1.1893	1.15230	0.78540	0.82107
$\bar {c_{t}}$	0.69319	1.0106	1.0106	1.1406	1.2099
$\sigma _{c_{t}}$	0.18291	0.17462	0.17752	0.27117	0.28089
*c*_*t*_ violation	477 (47.7 %)	15 (1.5 %)	18 (1.8 %)	23 (2.3 %)	13 (1.3 %)
*α*=1.65					
$\bar {J}$	6.5268	10.04	9.6011	8.2477	8.5514
*σ*_*J*_	0.57543	0.88430	0.83741	0.73621	0.76632
$\bar {c_{t}}$	0.69319	1.0143	1.0157	1.0339	1.0933
$\sigma _{c_{t}}$	0.18291	0.20620	0.21414	0.25176	0.26120
*c*_*t*_ violation	477 (47.7 %)	31 (3.1 %)	39 (3.9 %)	53 (5.3 %)	34 (3.4 %)

**Expected value and 95 % confidence bound** First the expected value and 95 % confidence bound of *S*_5_ (based on *α*=1.65) are compared. This is done in Figs. 8(e)-(f). From Fig. 8(e)-(f) it can be seen that the expected state and 95 % confidence bounds for *S*_5_ are similar for the PCE1 and PCE2 approaches. However, the linearization approach and sigma points approach take initially more distance from the nominal profile. In this case, similarly to Case1, a linear approximation technique (i.e., the linearization approach) is more conservative, implying less constraint violations. All 95 % confidence bounds are at 0.675 *mM* at the end as required by the imposed constraint in the implementation of the approximation techniques for uncertainty propagation.

Both PCE1 and PCE2 have a more accurate prediction of the expected value and variance of the objective function *J* and the terminal constraint function value *c*_*t*_ than the linearization and sigma points approach (in Table 6), when comparing with the empirically calculated expected values and variances with Monte Carlo simulations (in Table 7).

**Constraint violations** The percentage of constraint violations is investigated by performing a Monte Carlo simulation with 1000 noise realizations from the parametric uncertainty distribution. From these simulations, it is observed that all four methods reduce the amount of constraint violations significantly: from 47.7 % in the nominal case to even 1.3 % for PCE2, when a backoff parameter value of *α*=1.96 is chosen in case of a normal parametric uncertainty distribution. For this case study, the PCE2 method is superior in performance, when considering number of constraint violations.

**Parametric uncertainty distribution** For this case study, the integration of prior information on the parametric uncertainty distribution in the polynomial chaos expansion approaches is studied. The orthogonal polynomials for *K*_*m*_, *λ* and *k*_cat_ are derived via the definition of orthogonal polynomials. Details can be found in Additional file 4.

For a normal and uniform parametric uncertainty distribution, a Monte Carlo simulation procedure with 1000 noise realizations has been followed and the results are summarized in Tables 7 and 8.

**Table 8:** Case 2 - Results Monte Carlo simulations (*N*=1000) in case of three uniformly distributed uncertain parameters (*K*_*m*_,*λ* and *k*_cat_) for robustified terminal constraint and objective function with the number of constraint violations, mean values and variances on the objective function and terminal constraint, respectively

	Nominal case	Linearization	Sigma points	PCE1	PCE2	PCE2 uniform
*α*=1.96						
$\bar {J}$	6.5517	13.048	12.599	8.8045	9.1717	9.1498
*σ*_*J*_	0.5323	1.1015	1.0665	0.7270	0.7600	0.7600
$\bar {c_{t}}$	0.7023	1.0205	1.0208	1.1555	1.2254	1.2240
$\sigma _{c_{t}}$	0.1744	0.1659	0.1687	0.2590	0.2684	0.2690
*c*_*t*_ violation	470 (47.0 %)	10 (1.0 %)	13 (1.3 %)	21 (2.1 %)	7 (0.7 %)	9 (0.9 %)
*α*=1.65						
$\bar {J}$	6.5517	10.079	9.6385	8.2798	8.5848	8.5683
*σ*_*J*_	0.5323	0.8195	0.7762	0.6813	0.7092	0.7086
$\bar {c_{t}}$	0.7030	1.0259	1.0277	1.0476	1.1077	1.1056
$\sigma _{c_{t}}$	0.1744	0.1966	0.2042	0.2404	0.2495	0.2496
*c*_*t*_ violation	470 (47.0 %)	28 (2.8 %)	36 (3.6 %)	55 (5.5 %)	29 (2.9 %)	29 (2.9 %)

As in the first case study, the percentage of constraint violations is slightly higher when a uniform distribution is considered. For this case study, the difference in performance between a uniform parametric uncertainty distribution and a normal parametric uncertainty distribution for the polynomial chaos expansion, is small. Gathering information on the parametric uncertainty distribution from a parameter identification procedure is intensive and does not lead to a drastic improvement in performance for this case study.

## Conclusions

In this work parametric uncertainty has been taken into account for prediction and control of biological networks. A critical comparison of three approximation techniques for uncertainty propagation, i.e., the linearization, sigma points and polynomial chaos expansion approaches has been made for dynamic optimization of biological networks under parametric uncertainty. The main advantage of the polynomial chaos expansion is its ability to tackle more easily non-normal parametric uncertainty distributions. Two case studies are investigated: *(i)* the minimization of intemediate metabolite accumulation in a basic three-step linear pathway model (with 3 metabolites, 3 fluxes, 4 differential states and 3 controls) and *(ii)* the multi-objective optimization (i.e., the minimization) of the final time and enzymatic cost a glycolysis inspired network model (with 4 metabolites, 4 fluxes, 8 differential states and 4 controls). For further analysis of the robustness, emphasis was put on a single objective: in Case 1 the minimization of the intermediate accumulation and in Case 2 the minimization of the enzymatic cost. In a next step, the robustness of the optimal control profiles obtained with the different approximation techniques for uncertainty propagation is investigated. Monte Carlo simulations are used for the assessment of these control profiles. From the results for both case studies, the different uncertainty propagation strategies each offer a robust solution under parametric uncertainty. When making the trade-off between computation time and the robustness of the obtained profiles, the sigma points and polynomial chaos expansion strategies score the best. In both case studies the effect of taking a uniform probability distribution for the parametric uncertainty has been taken into account. However, the gain by taking a uniform probability distribution into account instead of a normal probability distribution is low. There is a reduction on the considered backoff and the variance of the objective functions and constraint. On the other hand, an upfront identification procedure for the parametric uncertainty distribution is time-consuming, expensive and does not offer a substantial advantage for the considered case studies in comparison with assuming a normal parametric uncertainty distribution. The linearization, sigma points and polynomial chaos expansion approaches offer a great potential for optimization and modeling under uncertainty in systems biology. The application of these approximation techniques for uncertainty propagation to large scale biological network models is the subject of future work. The integration of these approximation techniques for uncertainty propagation in an interactive tool for multi-objective dynamic optimization [43] is also part of the future work.

## Additional files

Additional file 1 Detailed review on approximation techniques for uncertainty propagation. A detailed review of the approximation techniques for uncertainty propagation: linearization, sigma points and polynomial chaos expansion approach. (PDF 479 kb)

Additional file 2 Optimization methods and software. A more in depth description of the used optimization methods and software. (PDF 242 kb)

Additional file 3 Numerical results Case 1. A detailed overview of the numerical results for Case 1. (PDF 667 kb)

Additional file 4 Numerical results Case 2. A detailed overview of the numerical results for Case 2. (PDF 708 kb)

## Abbreviations

CPU: Central processing unit
KKT: Karush-Kuhn-Tucker
LIN: Linearization
MC: Monte Carlo
NBI: Normal boundary intersection
NLP: Nonlinear programming problem
PCE: Polynomial chaos expansion
PCE1: First order polynomial chaos expansion
PCE2: Second order polynomial chaos expansion
SP: Sigma points
