# Supplementary material for: Dynamic optimization of biological networks under parametric uncertainty
Source: BMC Syst Biol. 2016 Aug 31;10:86. doi: 10.1186/s12918-016-0328-6 (PMC5006366; doi:10.1186/s12918-016-0328-6)
Supplement: Additional file 1 — Detailed review on approximation techniques for uncertainty propagation. A detailed review of the approximation techniques for uncertainty propagation: linearization, sigma points and polynomial chaos expansion approach. (PDF 479 kb) [file 12918_2016_328_MOESM1_ESM.pdf]

Additional file 1: Detailed review on approximation techniques for uncertainty propagation

Philippe Nimmegeers, Dries Telen, Filip Logist, Jan Van Impe

Introduction

In this Additional file a detailed review on three approximation techniques for uncertainty propagation is presented: linearization, sigma points and polynomial chaos expansion. To conclude an example of uncertainty propagation for 1 uncertain parameter is elaborated. This example allows to reveal some theoretical insights on the three uncertainty propagation techniques.

Linearization approach

Assume that the parametric uncertainty is characterized by a probability distribution with mean $\bar{\boldsymbol{\theta}}$ and variance-covariance matrix $\boldsymbol{\Sigma}$. It is assumed that from the result of a previous parameter estimation procedure, the current best guess for $\bar{\boldsymbol{\theta}}$ is the currently used parameter value $\boldsymbol{\theta}_{\text{nom}}$, to which is referred to as the *nominal parameter value* in the remainder of this text [1].

The variance-covariance matrix for the states $\mathbf{P}_{\text{LIN}}$ is computed by integrating the model equations together with the system's first order sensitivity equations with respect to the parameters. These sensitivity equations are presented in Equation (1).

$$\begin{cases} \dot{\mathbf{S}}_{\text{LIN}}(t) = \frac{\partial \mathbf{f}(\mathbf{x},\mathbf{u},\boldsymbol{\theta}_{\text{nom}},t)}{\partial \mathbf{x}} \mathbf{S}_{\mathbf{LIN}} + \frac{\partial \mathbf{f}(\mathbf{x},\mathbf{u},\boldsymbol{\theta}_{\text{nom}},t)}{\partial \boldsymbol{\theta}}, \\ \mathbf{S}_{\text{LIN}}(0) = \mathbf{0} \end{cases} \tag{1}$$

with $\mathbf{S}_{\text{LIN}}(t) = \frac{\partial \mathbf{x}}{\partial \theta}$ the sensitivities of the states with respect to the parameters. The states' variance-covariance matrix $\mathbf{Var}[\mathbf{x}]$ is linearly approximated by $\mathbf{P}_{\text{LIN}}$ as shown in Equation (2):

$$\mathbf{Var}[\mathbf{x}] \approx \mathbf{P}_{\text{LIN}} = \mathbf{S}_{\text{LIN}}(t) \boldsymbol{\Sigma} \mathbf{S}_{\text{LIN}}(t)^{\top}. \tag{2}$$

The propagation of the states' uncertainty is used to make the dynamic optimization problem more robust with respect to path and/or constraint violations or the objective.

The expected value of R_k is approximated with the linearization approach by:

$$\mathbf{E}[R_k] \approx R_k(\mathbf{x}, \mathbf{u}, \bar{\boldsymbol{\theta}}, t) \tag{3}$$

The variance-covariance matrix of the considered constraint or objective function R_k that is robustified, with the linearization approach is formulated as follows:

$$\begin{aligned} \mathbf{Var}[R_k] &\approx \mathbf{P}_{R_k R_k,\text{LIN}} \\ \mathbf{P}_{R_k R_k,\text{LIN}} &= \left(\frac{\partial R_k}{\partial \mathbf{x}}\right) \mathbf{P}_{\text{LIN}} \left(\frac{\partial R_k}{\partial \mathbf{x}}\right)^{\top} \end{aligned} \tag{4}$$

The robustification of R_k is done as shown in Equation (5).

$$R_{k,\text{LIN}} = R_k + \alpha_{R_k,\text{LIN}} \sqrt{\mathbf{P}_{R_k R_k,\text{LIN}}} \tag{5}$$

Since $n_x n_\theta$ additional states are needed for the sensitivity equations and the original problem has n_x states, the total number of states for the robustified optimization problem with the linearization approach equals $n_x n_\theta + n_x$.

A graphical representation of uncertainty propagation with the linearization approach is presented in Figure 1.

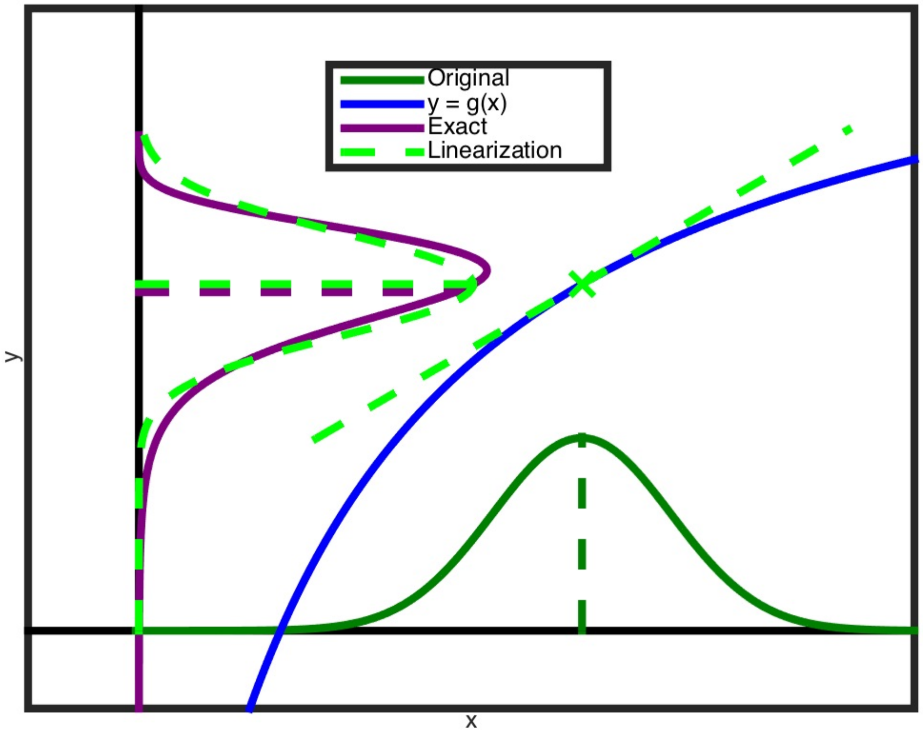

Figure 1: Graphical representation of uncertainty propagation with the linearization approach.

Sigma points approach

The sigma points approach [2] approximates nonlinear transformations of probability distributions. The sigma points approach is applicable to any symmetric, unimodal parametric uncertainty distribution (e.g., normal distribution). This approach approximates a probability distribution with a fixed number of parameters, i.e., the so-called *sigma points*, rather than approximating an arbitrary nonlinear function. There is no unique way to choose these sigma points. Several methods, each with their advantages have been proposed in, e.g., [3].

Assume that the current best guess for the model parameters is the nominal parameter vector

$\boldsymbol{\theta}_{\text{nom}}$ with variance-covariance $\boldsymbol{\Sigma}$, then the *sigma points* are the following:

$$\begin{aligned}
&\boldsymbol{\pi}_0 = \boldsymbol{\theta}_{\text{nom}} \ , \\
&\boldsymbol{\pi}_i = \boldsymbol{\theta}_{\text{nom}} + \sqrt{(n_\theta + \kappa)\boldsymbol{\Sigma}}_i \\
&\text{with } i = 1, \ldots, n_\theta \ , \\
&\boldsymbol{\pi}_i = \boldsymbol{\theta}_{\text{nom}} - \sqrt{(n_\theta + \kappa)\boldsymbol{\Sigma}}_{i-n_\theta} \\
&\text{with } i = n_\theta + 1, \ldots, 2n_\theta \ .
\end{aligned}$$

with κ a term for including knowledge regarding higher moments of the given probability distribution (and chosen as $\kappa = 3 - n_\theta$ to minimize the mean squared error up to fourth order [2]) and $\sqrt{\boldsymbol{\Sigma}}_i$, the i-th column of the parameter variance-covariance matrix square root which can be computed by, e.g., a Cholesky decomposition.

The model has to be evaluated $2n_\theta + 1$ times in order to approximate the distribution of the model states. These evaluations only differ in the parameter values, i.e., the *sigma points*.

The predicted expected value of R_k is computed in the sigma points method as:

$$\begin{aligned}
&\mathbf{E}\left[R_k\right] \approx \bar{R}_{k,\text{SP}} \\
&\bar{R}_{k,\text{SP}} = \frac{1}{n_\theta + \kappa}\left(\kappa R_k(\boldsymbol{\pi}_0) + \frac{1}{2}\sum_{i=1}^{2n_\theta} R_k(\boldsymbol{\pi}_i)\right) \ ,
\end{aligned} \tag{6}$$

while the predicted variance-covariance matrix is formulated as:

$$\begin{aligned}
\mathbf{Var}\left[R_k\right] &\approx \mathbf{P}_{\text{R}_\text{k}\text{R}_\text{k},\text{SP}} \\
&= \frac{\left(\kappa(R_k(\boldsymbol{\pi}_0) - \bar{R}_k)(R_k(\boldsymbol{\pi}_0) - \bar{R}_k)^\top\right)}{n_\theta + \kappa} \\
&+ \frac{\left(\frac{1}{2}\sum_{i=1}^{2n_\theta}(R_k(\boldsymbol{\pi}_i) - \bar{R}_k)(R_k(\boldsymbol{\pi}_i) - \bar{R}_k)^\top\right)}{n_\theta + \kappa} \ .
\end{aligned} \tag{7}$$

This robustification of R_k is done as shown in Equation (8).

$$R_{k,\text{SP}} = \bar{R}_{k,\text{SP}} + \alpha_{R_k,\text{SP}}\sqrt{\mathbf{P}_{\text{R}_\text{k}\text{R}_\text{k},\text{SP}}} \tag{8}$$

The total amount of states to be computed in the sigma points approach equals $(2n_\theta + 1)n_x$. Since only the parameter values differ between the different sigma points, the procedure is easily parallelized. There is no significant difference in the number of states (i.e., $n_x n_\theta$), when compared with the linearization approach.

A graphical representation of uncertainty propagation with the sigma points approach is presented in Figure 2.

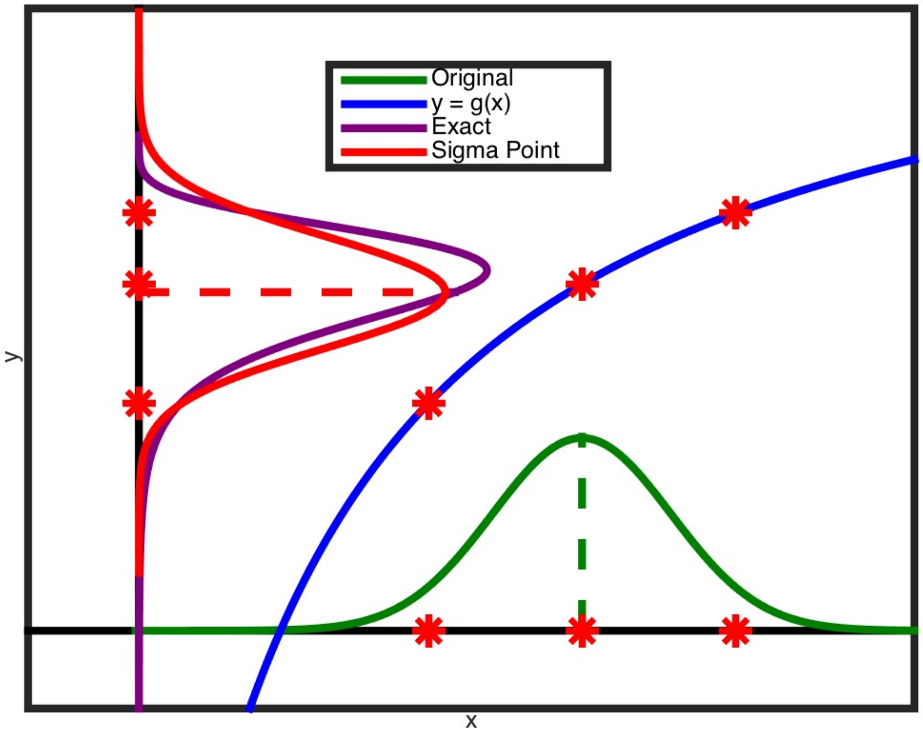

Figure 2: Graphical representation of uncertainty propagation with the sigma points approach.

Polynomial chaos expansion approach.

The origin of the polynomial chaos expansion (PCE) approach lies in performing an uncertainty analysis on large complex models. The rationale of this approach is to approximate a model response as a sum of orthogonal polynomials (i.e., polynomials of which the inner product equals zero) through *PCE sampling points* [4]. These polynomials are a function of the uncertain parameters for which a probability distribution is given. These orthogonal polynomials can be based on the Wiener-Askey scheme [5] or derived from the definition of orthogonal polynomials [4]. Orthogonal polynomials are defined as follows:

$$\int_{-\infty}^{+\infty} \rho(\theta_{i_\theta})\phi_i(\theta_{i_\theta})\phi_j(\theta_{i_\theta})d\theta_{i_\theta} = 0 \quad \text{for } i \neq j. \tag{9}$$

$\rho(\theta_{i_\theta})$ represents the probability density function characterizing the parametric uncertainty, while $\phi_i(\theta_{i_\theta})$ and $\phi_j(\theta_{i_\theta})$ are the univariate orthogonal polynomials of order i and j respectively and θ_{i_θ} is an uncertain parameter. Since $\phi_0(\theta_{i_\theta}) = 1$ by definition, all the higher order orthogonal polynomials can be derived with the definition in Equation (9).

Consider the constraint or objective function that is robustified R_k. The polynomial chaos expansion $R^{(p)}_{k,\mathrm{PCE}}$ of order p of the robustified constraint or objective function R_k is defined in Equation (10):

$$R^{(p)}_{k,\mathrm{PCE}} = a^{(p)}_{R_k,0} + \sum_{i_1=1}^{n_\theta} a^{(p)}_{R_k,i_1}\psi_1(\theta_{i_1}) + \sum_{i_1=1}^{n_\theta}\sum_{i_2=1}^{i_1} a^{(p)}_{R_k,i_1 i_2}\psi_2(\theta_{i_1}\theta_{i_2}) \\ + \sum_{i_1=1}^{n_\theta}\sum_{i_2=1}^{i_1}\sum_{i_3=1}^{i_2} a^{(p)}_{R_k,i_1 i_2 i_3}\psi_3(\theta_{i_1},\theta_{i_2},\theta_{i_3}) + \ldots \tag{10}$$

with $\psi_d(\theta_{i_1}\ldots\theta_{i_d})$ the multivariate orthogonal polynomial of order $d = i_1, i_2, \ldots, i_{n_\theta}$ that equals the product of univariate orthogonal polynomials $\phi_i(\theta_{i_\theta})$ and $a^{(p)}_{R_k,0}$, $a^{(p)}_{R_k,i_1}$ and $a^{(p)}_{R_k,i_1\ldots i_{n_\theta}}$ unknown PCE coefficients for R_k, with $k = 1, \ldots, n_R$ an index running over the n_R robustified variables.

The total number of terms L in the polynomial chaos expansion of order p depends on the number of uncertain parameters n_θ and the order of the expansion p [6]:

$$L = \frac{(n_\theta + p)!}{n_\theta! p!}. \tag{11}$$

For notation the PCE expansion is reformulated with a term based index j ($j = 0, \ldots, L$):

$$R^{(p)}_{k,\mathrm{PCE}}(\boldsymbol{\theta}) = \sum_{j=0}^{L-1} a^{(p)}_{R_k,j}\Phi_j(\boldsymbol{\theta}), \tag{12}$$

with $a^{(p)}_{R_k,j}$ the unknown PCE coefficients and $\Phi_j(\boldsymbol{\theta})$ the multivariate orthogonal polynomials and j a term based index ($j = 0, \ldots, L$).

In order to determine the unknown coefficients $a^{(p)}_{R_k,j}$, a non-intrusive PCE rationale is followed: the model is evaluated in *sampling points*, which results in the vector $\mathbf{R}_{\mathbf{k},\mathrm{s}} = \begin{bmatrix} R_k(\boldsymbol{\pi}_\mathbf{0}) & \ldots & R_k(\boldsymbol{\pi}_{\boldsymbol{n_s}-\mathbf{1}})\end{bmatrix}^\top$ containing R_k at each sampling point and that R_k equals its polynomial chaos expansion in each sampling point. The sampling points are selected from the roots of the higher order (i.e., $p+1$) orthogonal polynomial for each uncertain parameter. Since there are more roots of those polynomials than there are necessary *sampling points* to determine the unknown coefficients $a^{(p)}_{R_k,j}$, parameter sets that span the high probability regions of their distributions are selected [4]. This leads to a system of n_s linear equations and L unknown coefficients $a^{(p)}_{R_k,j}$ as described in Equation (13).

$$\mathbf{R}_{\mathbf{k},\mathrm{s}} = \left(\boldsymbol{\Lambda}^{(p)}\right)^\top \mathbf{a}_{\mathbf{R_k}}{}^{(p)}, \tag{13}$$

with $\mathbf{a}_{\mathbf{R_k}}{}^{(p)} = \begin{bmatrix} a^{(p)}_{R_k,0} & \ldots & a^{(p)}_{R_k,L-1}\end{bmatrix}^\top$, $\boldsymbol{\pi}_i$ the parameter vector corresponding to the ith *sampling point*, $\mathbf{R}^{(p)}_{\mathbf{k},\mathrm{s}} \in \mathbb{R}^{n_s}$ the vector containing R_k evaluated at the *sampling points* $\boldsymbol{\pi_i}$, n_s the number of *sampling points* ($n_s \geq L$) and $\boldsymbol{\Lambda}^{(p)} \in \mathbb{R}^{L\times n_s}$ presented in Equation (14).

$$\boldsymbol{\Lambda}^{(p)} = \begin{bmatrix} \Phi_0(\boldsymbol{\pi}_\mathbf{0}) & \ldots & \Phi_0(\boldsymbol{\pi}_{\mathbf{n_s}-\mathbf{1}}) \\ \vdots & \ddots & \vdots \\ \Phi_{L-1}(\boldsymbol{\pi}_\mathbf{0}) & \ldots & \Phi_{L-1}(\boldsymbol{\pi}_{\mathbf{n_s}-\mathbf{1}}) \end{bmatrix} \tag{14}$$

Since $\mathbf{\Lambda}^{(p)}$ only depends on the *sampling points* and the orthogonal polynomials, this matrix is known upfront. Therefore, the coefficients $a^{(p)}_{R_k,j}$ in $\mathbf{a_{R_k}}^{(p)}$ can be calculated explicitly with the following least squares approximation of the system in Equation (13) [7]:

$$\mathbf{a_{R_k}}^{(p)} = \left(\mathbf{\Lambda}^{(p)}\left(\mathbf{\Lambda}^{(p)}\right)^{\top}\right)^{-1}\mathbf{\Lambda}^{(p)}\mathbf{R}_{\mathbf{k},\mathrm{s}}. \tag{15}$$

The coefficients of the polynomial chaos expansion in $\mathbf{a_{R_k}}^{(p)}$ are computed as a weighting of the *sampling points* $\mathbf{R}^{(p)}_{\mathbf{k},\mathrm{s}}$ with $\left(\mathbf{\Lambda}^{(p)}\left(\mathbf{\Lambda}^{(p)}\right)^{\top}\right)^{-1}\mathbf{\Lambda}^{(p)}$ as weighting matrix in Equation (15). A necessary condition for using this explicit approach is that the matrix $\mathbf{\Lambda}^{(p)}\left(\mathbf{\Lambda}^{(p)}\right)^{\top}$ is nonsingular. This least squares approximation in Equation (15) is only needed, if more *PCE sampling points* are used for the polynomial chaos expansion than there are coefficients (i.e., $n_s > L$). Else (i.e., for $n_s = L$), the formulation is simplified to: $\mathbf{a_{R_k}}^{(p)} = \left(\left(\mathbf{\Lambda}^{(p)}\right)^{\top}\right)^{-1}\mathbf{R}^{(p)}_{\mathbf{k},\mathrm{s}}$. The coefficients $\mathbf{a_{R_k}}^{(p)}$ of the polynomial chaos expansion are calculated during the optimization and are time dependent.

Different conditions that can be checked before the optimization, have to be satisfied such that the *sampling points* can be used for the polynomial chaos expansion. These conditions are formulated in general for if one would use more (or as much) *sampling points* than (as) there are terms in the polynomial chaos expansion (i.e., $n_s > L$):

1. $\mathbf{\Lambda}^{(p)}\left(\mathbf{\Lambda}^{(p)}\right)^{\top}$ is invertible,
2. $\mathbf{C} = \left(\mathbf{\Lambda}^{(p)}\left(\mathbf{\Lambda}^{(p)}\right)^{\top}\right)^{-1}\mathbf{\Lambda}^{(p)}$ has full rank.

The expected value and variance of R_k are approximated with the polynomial chaos expansion approach as:

$$\mathbf{E}\left[R_k\right] \approx \bar{R}^{(p)}_{k,PCE} = a^{(p)}_{R_k,0} \tag{16}$$

$$\mathbf{Var}\left[R_k\right] \approx \mathbf{P}^{(p)}_{\mathrm{R_kR_k,PCE}} = \sum_{j=1}^{L-1}\left(a^{(p)}_{R_k,j}\right)^2\mathbf{E}\left[\Phi_j^2(\boldsymbol{\theta})\right] \tag{17}$$

The authors want to stress that $\mathbf{E}\left[\Phi_j^2(\boldsymbol{\theta})\right]$ can be computed offline and upfront.

When standard normalized polynomials (e.g., standard Hermite polynomials) are employed, the variance computation reduces to Equation (18) [8].

$$\mathbf{P}^{(p)}_{\mathrm{R_kR_k,PCE}} = \sum_{j=1}^{L-1}\left(a^{(p)}_{R_k,j}\right)^2 \tag{18}$$

The robustification of R_k with the polynomial chaos expansion method is then as in Equation (19).

$$R^{(p)}_{k,\mathrm{PCE}} = \bar{R}^{(p)}_{k,\mathrm{PCE}} + \alpha_{R_k,\mathrm{PCE}}\sqrt{\mathbf{P}^{(p)}_{\mathrm{R_kR_k,PCE}}} \tag{19}$$

In this work a first order (PCE1) and second order (PCE2) polynomial chaos expansion approach are investigated. This results in a number of $(n_\theta + 1)n_x$ for PCE1 and $\frac{(n_\theta+2)(n_\theta+1)}{2} = \frac{(n_\theta^2+3n_\theta+2)}{2}$ states for PCE2.

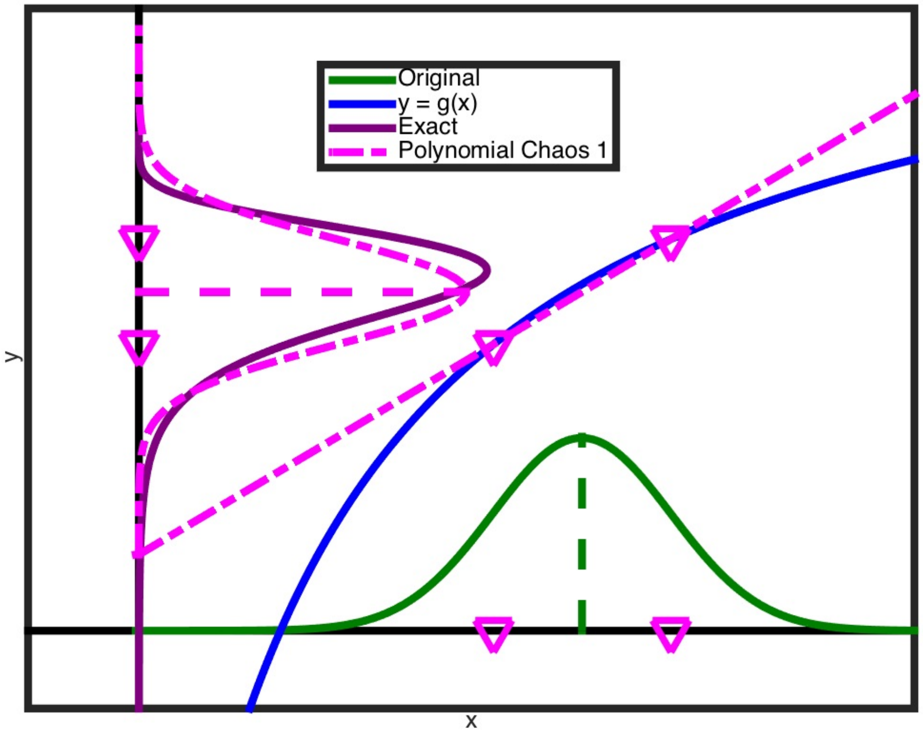

Figure 3: Graphical representation of uncertainty propagation with the first order polynomial chaos expansion approach.

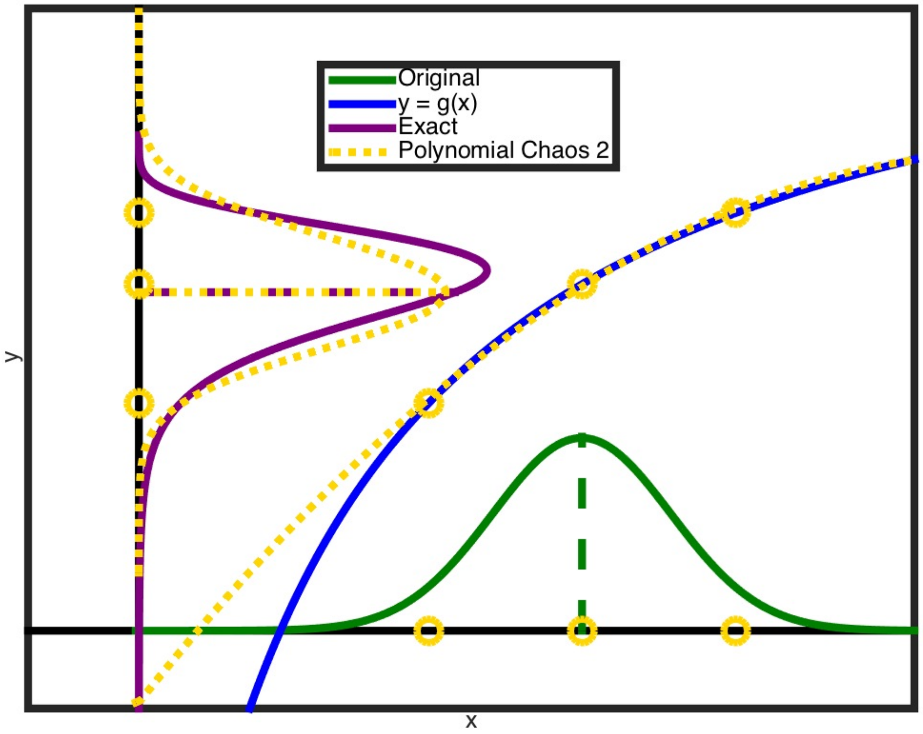

Figure 4: Graphical representation of uncertainty propagation with the second order polynomial chaos expansion approach.

Comparison of the uncertainty propagation techniques

The polynomial chaos expansion approach has some similarities with the sigma points approach [2]. The main differences are how the *sampling points* $\boldsymbol{\pi}_\mathbf{i}$ are selected, the amount of *sampling points*, the calculation of the moments and the number of additional equations. The PCE1 approach has the same number of states as the linearization approach. The PCE2 approach has $(\frac{n_\theta^2}{2} + \frac{n_\theta}{2})n_x$ more states than the linearization approach and $(\frac{n_\theta^2}{2} - \frac{n_\theta}{2})n_x$ more states than the sigma points approach. If only one uncertain parameter is considered, the sigma points approach and PCE2 approach have the same number of states.

Example: Uncertainty propagation for 1 uncertain parameter

First the formulations for the expected value and variance-covariance matrix are presented for the generic case of a nonlinear function $y = g(x)$. Subsequently a numerical example is elaborated, which was the nonlinear function used for the illustrations of the approximation techniques for uncertainty propagation.

General nonlinear function

Consider the example of the nonlinear model $y = g(x)$, in Figure 5. The parameter x is considered as the only uncertain parameter with a normal parametric uncertainty distribution with expected value x and standard deviation σ_x. Applying the three uncertainty propagation techniques to this example, reveals some theoretical insights. The expected value approximations, obtained with the uncertainty propagation techniques are presented in Table 1.

Table 1: Expected value for $y = g(x)$ with one uncertain parameter x for linearization, sigma points, PCE1 and PCE2

	Linearization	Sigma Points	PCE1	PCE2
$\mathbf{E}[y]$	$g(x)$	$\frac{2}{3}g(x) + \frac{1}{6}g(x + \sqrt{3})\sigma_x + \frac{1}{6}g(x - \sqrt{3}\sigma_x)$	$\frac{1}{2}g(x + \sigma_x) + \frac{1}{2}g(x - \sigma_x)$	$\frac{2}{3}g(x) + \frac{1}{6}g(x + \sqrt{3}\sigma_x) + \frac{1}{6}g(x - \sqrt{3}\sigma_x)$

From this table it is clear that the sigma points approach and PCE2 approach have the same way of approximating the expected value, when considering the weights. This is the case for one uncertain parameter, but also for the case studies with 3 uncertain parameters in this work.

The propagated uncertainty distribution of y is shown in Figure 5. The propagated uncertainty distributions for y calculated with the sigma points approach and PCE2 approach are clearly coinciding. It can also be proven that the variance calculation is the same for the sigma points approach and PCE2 approach, for one uncertain parameter and a normal parametric uncertainty distribution.

Since only those two statistical moments are used, the second order polynomial chaos expansion boils down to the sigma points approach when only one parameter is uncertain and a non-intrusive polynomial chaos expansion approach is applied.

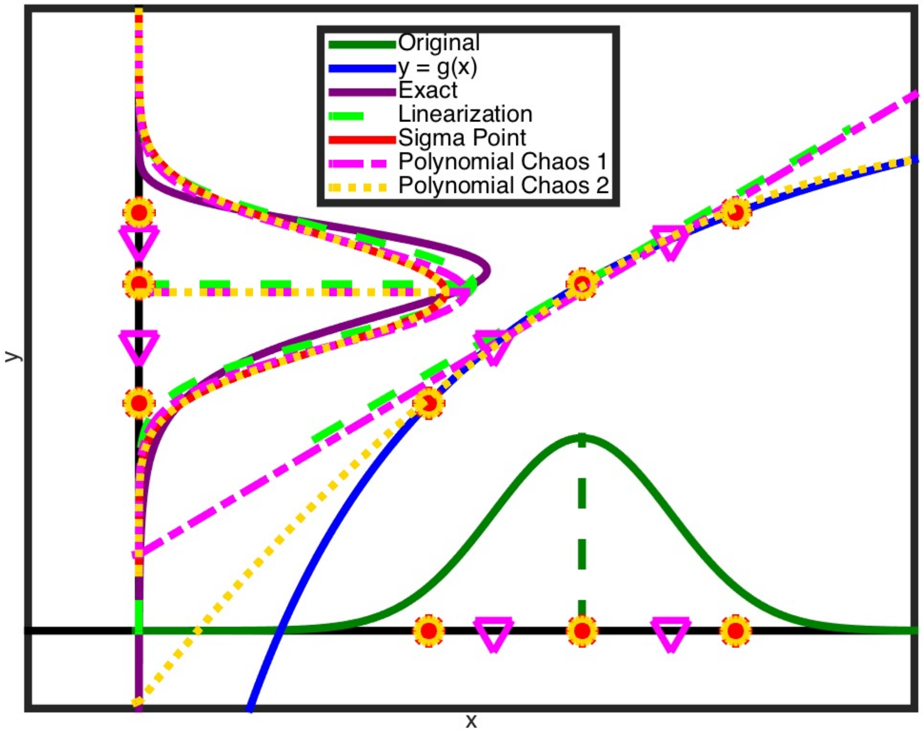

Figure 5: Overview of the linearization, sigma points and polynomial chaos expansion methods for uncertainty propagation of x towards $y = g(x)$.

Numerical example

As a numerical example and for the generation of the figures the three approximation techniques for uncertainty propagation are elaborated for the following nonlinear function:

$$y(x) = 9 - \frac{5.0}{(x + 0.3)} \tag{20}$$

It is assumed that x is the only uncertain parameter, following a normal distribution with mean value x_{nom} and standard deviation 0.1. For the remainder of this example $x_{\text{nom}} = 1$

Linearization approach

For the linearization approach this means that the expected value of $y(x)$ equals:

$$\mathbf{E}[y] \approx \bar{y}_{LIN} = 9 - \frac{5.0}{(x_{\text{nom}} + 0.3)} \tag{21}$$

$$= 9 - \frac{5.0}{(x_{nom} + 0.3)} \tag{22}$$

$$= 5.1538 \tag{23}$$

As stated in (2) the states' variance-covariance matrix $\mathbf{Var}[\mathbf{x}]$ is linearly approximated by $\mathbf{P}_{\text{LIN}}$, which equals:

$$\mathbf{Var}[\mathbf{x}] \approx \mathbf{P}_{\text{LIN}} = \mathbf{S}_{\text{LIN}}(t)\boldsymbol{\Sigma}\mathbf{S}_{\text{LIN}}(t)^{\top}. \tag{24}$$

$$= 0.01 \tag{25}$$

This is also logical, since the only considered parameter in this example is x.

The variance-covariance matrix is approximated with the linearization approach by:

$$\mathbf{Var}[y] \approx \mathbf{P}_{yy,\text{LIN}} = \left(\frac{\partial y}{\partial x}\right) \mathbf{P}_{\text{LIN}} \left(\frac{\partial y}{\partial \mathbf{x}}\right)^{\top} \tag{26}$$

$$= \left(\frac{5}{(x_{\text{nom}} + 0.3)^2}\right) 0.01 \left(\frac{5}{(x_{\text{nom}} + 0.3)^2}\right)^{\top} \tag{27}$$

$$= 0.0875 \tag{28}$$

Sigma points approach

For this numerical example, there are three sigma points:

$$\pi_0 = x_{nom} = 1 \tag{29}$$

$$\pi_1 = x_{nom} + 0.1\sqrt{3} = 1 + 0.1\sqrt{3} \tag{30}$$

$$\pi_2 = x_{nom} - 0.1\sqrt{3} = 1 - 0.1\sqrt{3} \tag{31}$$

$$\tag{32}$$

Since there is only 1 uncertain parameter, $\kappa = 2$. The expected value of y is therefore approximated with the sigma points approach as:

$$\mathbf{E}[y] \approx \bar{y}_{\text{SP}} = \frac{1}{n_\theta + \kappa}\left(\kappa R_k(\boldsymbol{\pi}_0) + \frac{1}{2}\sum_{i=1}^{2n_\theta} R_k(\boldsymbol{\pi}_i)\right), \tag{33}$$

$$= \frac{1}{3}\left(2y(x_{\text{nom}}) + \frac{1}{2}\left((y(x_{nom} + 0.1\sqrt{3}) + y(x_{nom} - 0.1\sqrt{3})\right)\right), \tag{34}$$

$$= \frac{2}{3}y(x_{\text{nom}}) + \frac{1}{6}y(x_{nom} + 0.1\sqrt{3}) + \frac{1}{6}y(x_{\text{nom}} - 0.1\sqrt{3}) \tag{35}$$

$$= 5.1307 \tag{36}$$

For the variance-covariance matrix approximation of y Equation (37) holds.

$$\begin{aligned}\mathbf{Var}[y] \approx \mathbf{P}_{\mathrm{yy,SP}} \\ &= \frac{\left(2(y(x_{\mathrm{nom}})-\bar{y})(y(x_{\mathrm{nom}})-\bar{y})^\top\right)}{3} + \frac{\left(\frac{1}{2}\sum_{i=1}^{2}(y(\pi_i)-\bar{y})(y(\pi_i)-\bar{y})^\top\right)}{3}. \\ &= 0.0918 \end{aligned} \tag{37}$$

First order polynomial chaos expansion approach

First a first order polynomial chaos expansion is calculated. Since a normal parametric uncertainty is assumed for x, normalized Hermite polynomials are applied.

Table 2: Normalized Hermite polynomials up till third order with ξ a standard normally distributed variable.

Order	Hermite	Roots
0	1	-
1	ξ	0
2	$\frac{1}{\sqrt{2}}(\xi^2-1)$	-1, $+1$
3	$\frac{1}{\sqrt{6}}(\xi^3-3\xi)$	$-\sqrt{3}$, 0, $\sqrt{3}$

A first order polynomial chaos expansion for 1 uncertain parameter is given by Equation (39)

$$y_{PCE1} = a_{y,0}^{(1)} + a_{y,1}^{(1)}\xi \tag{38}$$

The sampling points for the first order polynomial chaos expansion are based on the roots r_1 and r_2 of the second order orthogonal polynomial , i.e., -1 and $+1$, respectively. The two PCE1 sampling points ($L = \frac{(n_\theta+p)!}{n_\theta!p!} = 2$) are:

$$\pi_{0,\mathrm{PCE1}} = x_{nom} + \sigma_{nom} r_1 = 0.9 \tag{39}$$

$$\pi_{1,\mathrm{PCE1}} = x_{nom} + \sigma_{nom} r_2 = 1.1 \tag{40}$$

These sampling points are then entered in Equation (39). The polynomial chaos expansion should equal the output in the sampling points. This results into a system of two equations to determine the PCE1 coefficients $a_{y,0}^{(1)}$ and $a_{y,1}^{(1)}$.

$$y(\pi_0) \approx y_{PCE1}(\pi_0) = a_{y,0}^{(1)} - a_{y,1}^{(1)} \tag{41}$$

$$y(\pi_1) \approx y_{PCE1}(\pi_1) = a_{y,0}^{(1)} + a_{y,1}^{(1)} \tag{42}$$

For this case study the system is straightforward to solve and an explicit expression for the PCE1 coefficients can easily be obtained. However, when the number of uncertain parameters increases, this is not longer the case. Therefore, the matrix notation is elaborated here (albeit not necessary for this case study).

$$\mathbf{y}_{,\mathrm{s}} = \left(\mathbf{\Lambda}^{(1)}\right)^{\top} \mathbf{a_y}^{(1)}, \tag{43}$$

$$\tag{44}$$

with: $\mathbf{y}_{\mathrm{s}} = \begin{bmatrix} y(\pi_0) \\ y(\pi_1) \end{bmatrix}$, $\mathbf{a_y}^{(1)} = \begin{bmatrix} a_{y,0}^{(1)} \\ a_{y,1}^{(1)} \end{bmatrix}$ and $\left(\mathbf{\Lambda}^{(1)}\right)^{\top} = \begin{bmatrix} 1 & -1 \\ 1 & 1 \end{bmatrix}$.

An explicit expression for the PCE1 coefficients can be derived and the expected value and variance of y are approximated as:

$$\mathbf{E}[y] \approx \bar{y}_{PCE}^{(1)} = a_{y,0}^{(1)} \tag{45}$$

$$= \frac{y(\pi_0) + y(\pi_1)}{2} \tag{46}$$

$$= 5.1310 \tag{47}$$

$$\mathbf{Var}[R_k] \approx \mathbf{P}_{\mathrm{yy,PCE}}^{(1)} = \sum_{j=1}^{L-1} \left(a_{y,j}^{(1)}\right)^2 \tag{48}$$

$$= \left(a_{y,1}^{(1)}\right)^2 \tag{49}$$

$$= \left(\frac{y(\pi_1) - y(\pi_0)}{2}\right)^2 \tag{50}$$

$$= 0.0886 \tag{51}$$

Second order polynomial chaos expansion approach

The second order polynomial chaos expansion of y is expressed as

$$y_{PCE2} = a_{y,0}^{(2)} + a_{y,1}^{(2)}\xi + a_{y,2}^{(2)}\frac{1}{\sqrt{2}}(\xi^2 - 1) \tag{52}$$

The sampling points for the first order polynomial chaos expansion are based on the roots r_1, r_2 and r_3 of the third order orthogonal polynomial, i.e., $-\sqrt{3}$, 0 and $\sqrt{3}$, respectively. The three PCE2 sampling points ($L = \frac{(n_\theta+p)!}{n_\theta! p!} = 3$) are the same as the sigma points:

$$\pi_0 = x_{nom} = 1 \tag{53}$$

$$\pi_1 = x_{nom} - 0.1\sqrt{3} = 1 - 0.1\sqrt{3} \tag{54}$$

$$\pi_2 = x_{nom} + 0.1\sqrt{3} = 1 + 0.1\sqrt{3} \tag{55}$$

$$\tag{56}$$

As stated earlier, a system of equations is derived based on the definition of the polynomial chaos expansion to calculate the PCE coefficients.

$$y(\pi_0) \approx y_{PCE2}(\pi_0) = a_{y,0}^{(2)} - a_{y,1}^{(2)} \tag{57}$$

$$y(\pi_1) \approx y_{PCE2}(\pi_1) = a_{y,0}^{(2)} + a_{y,1}^{(2)} \tag{58}$$

$$y(\pi_2) \approx y_{PCE2}(\pi_2) = a_{y,0}^{(2)} + a_{y,1}^{(2)} \tag{59}$$

In matrix notation the system is formulated as:

$$\mathbf{y}_{,\mathrm{s}} = \left(\mathbf{\Lambda}^{(2)}\right)^{\top} \mathbf{a_y}^{(2)}, \tag{60}$$

$$\tag{61}$$

with: $\mathbf{y}_{\mathrm{s}} = \begin{bmatrix} y(\pi_0) \\ y(\pi_1) \\ y(\pi_3) \end{bmatrix}$, $\mathbf{a_y}^{(2)} = \begin{bmatrix} a_{y,0}^{(1)} \\ a_{y,1}^{(1)} \end{bmatrix}$ and $\left(\mathbf{\Lambda}^{(1)}\right)^{\top} = \begin{bmatrix} 1 & 0 & -1/sqrt(2) \\ 1 & -sqrt(3) & sqrt(2) \\ 1 & sqrt(3) & sqrt(2) \end{bmatrix}$.

This results into the following approximations for the expected value and variance with the PCE2 approach:

$$\begin{aligned}
\mathbf{E}[y] \approx \bar{y}_{PCE}^{(2)} &= a_{y,0}^{(2)} && (62)\\
&= \frac{2}{3} y(x_{\mathrm{nom}}) + \frac{1}{6} y(x_{nom} - 0.1\sqrt{3}) + \frac{1}{6} y(x_{\mathrm{nom}} + 0.1\sqrt{3}) && (63)\\
&= 5.1307 && (64)\\
\mathbf{Var}[R_k] \approx \mathbf{P}_{\mathrm{yy,PCE}}^{(2)} &= \sum_{j=1}^{3-1} \left(a_{y,j}^{(2)}\right)^2 && (65)\\
&= \left(a_{y,1}^{(2)}\right)^2 + \left(a_{y,2}^{(2)}\right)^2 && (66)\\
&= \frac{1}{12} \left(y(\pi_2) - y(\pi_1)\right)^2 + \left(\frac{\sqrt{2}}{6} y(\pi_1) + \frac{\sqrt{2}}{6} y(\pi_2) - \frac{\sqrt{2}}{3} y(\pi_0)\right)^2 && (67)\\
&= \frac{1}{12} \left(y(x_{\mathrm{nom}} + 0.1\sqrt{3}) - y(x_{\mathrm{nom}} - 0.1\sqrt{3})\right)^2 && (68)\\
&+ \left(\frac{\sqrt{2}}{6} y(x_{\mathrm{nom}} - 0.1\sqrt{3}) + \frac{\sqrt{2}}{6} y(x_{\mathrm{nom}} + 0.1\sqrt{3}) - \frac{\sqrt{2}}{3} y(x_{\mathrm{nom}})\right)^2 && (69)\\
&= 0.0918 && (70)
\end{aligned}$$

These results are summarized in Table 3. The exact distribution is calculated via a numerical integration using the trapezoidal rule over the parameter space.

Table 3: Calculated expected value and variance of the response y, computed with numerical integration, linearization, sigma points, first and second order polynomial chaos expansion for $x = 1$ and $sigma_x = 0.1$.

	Exact	Linearization	Sigma Points	PCE1	PCE2
Expected Value	5.1307	5.1538	5.1307	5.1310	5.1307
Variance	0.0919	0.0875	0.0918	0.0886	0.0918

For the generation of Figure 5 0.8 has been taken as nominal value for x and a standard deviation of 0.16 was assumed. This leads to the results in Table 4.

Table 4: Calculated expected value and variance of the response y, computed with numerical integration, linearization, sigma points, first and second order polynomial chaos expansion for $x = 0.8$ and $sigma_x = 0.16$.

	Exact	Linearization	Sigma Points	PCE1	PCE2
Expected Value	4.3515	4.4545	4.3519	4.3563	4.3519
Variance	0.5285	0.4372	0.5194	0.4562	0.5194

As it can be seen in Figure 5, the sigma points approach and PCE2 approach approximate the expected value and variance-covariance matrix of y in the same way and are the closest to the integration of the parameter space, when one parameter is considered to be uncertain.

Bibliography

[1] Srinivasan, B., Bonvin, D., Visser, E., Palanki, S.: Dynamic optimization of batch processes: II. role of measurements in handling uncertainty. Computers & Chemical Engineering **27**(1), 27–44 (2003)

[2] Julier, S., Uhlmann, J.K.: A general method for approximating nonlinear transformations of probability distributions. Departement of Chemical Engineering Science, University of Oxford (1996)

[3] Julier, S.J.: The Scaled Unscented Transformation, vol. 6, pp. 4555–4559 (2002)

[4] Webster, M.D., Tatang, M.A., McRae, G.J.: Application of the Probabilistic Collocation Method for an Uncertainty Analysis of a Simple Ocean Model. Report (MIT Joint Program on the Science and Policy of Global Change), (1996)

[5] Xiu, D., Karniadakis, G.E.: The wiener-askey polynomial chaos for stochastic differential equations. SIAM Journal of Scientific Computation **24**, 619–644 (2002)

[6] Mesbah, A., Streif, S., Findeisen, R., Braatz, R.D.: Stochastic nonlinear model predictive control with probabilistic constraints. In: American Control Conference (ACC), 2014, pp. 2413–2419 (2014)

[7] Garcia-Cabrejo, O., Valocchi, A.: Global sensitivity analysis for multivariate output using polynomial chaos expansion. Reliability Engineering and System Safety **126**, 25–36 (2014)

[8] Poles, S., Lovison, A.: A polynomial chaos approach to robust multiobjective optimization. In: Deb, K., Greco, S., Miettinen, K., Zitzler, E. (eds.) Hybrid and Robust Approaches to Multiobjective Optimization. Dagstuhl Seminar Proceedings (2009)
