# Supplementary material for: Dynamic optimization of biological networks under parametric uncertainty
Source: BMC Syst Biol. 2016 Aug 31;10:86. doi: 10.1186/s12918-016-0328-6 (PMC5006366; doi:10.1186/s12918-016-0328-6)
Supplement: Additional file 2 — Optimization methods and software. A more in depth description of the used optimization methods and software. (PDF 242 kb) [file 12918_2016_328_MOESM2_ESM.pdf]

Additional file 2: Optimization methods and software

Philippe Nimmegeers, Dries Telen, Filip Logist, Jan Van Impe

Multi-objective optimization methods

In practice, multiple objectives, which are very often conflicting with each other, have to be considered simultaneously, e.g., minimizing the enzyme consumption while maximizing the production of a certain metabolite. Therefore, a single optimal solution will not exist, but a set of *trade-off solutions*, called the Pareto front, is obtained when solving a multi-objective problem [1].

Two categories of methods can be distinguished for the calculation of Pareto fronts: *scalarization* methods (e.g., Weighted Sum [2],[3], ϵ-constraint [4], (Enhanced) Normalized Normal Constraint ((E)NNC) [5],[6] and Normal Boundary Intersection (NBI) [1],[7]) and *vectorization* methods [8], (e.g., MO Particle Swarm Optimization [9], MO Simulated Annealing [10], MO Genetic Algorithms [8]) [1]. The methods from the former category convert the multi-objective optimization problem into a series of single objective optimization problems by using scalar variables. Scalarization methods can take advantage of fast and efficient gradient based methods to find an optimum for the series of single objectives. Vectorization methods on the other hand start from a population of candidate solutions that gradually evolve to the Pareto front and as such tackle the multi-objective optimization problem directly. Vectorization methods often use derivative-free optimization methods as evolutionary or stochastic optimization approaches, which do not need gradients. However, the model equations are repeatedly solved in these methods, such that the computation time increases for large-scale models. Furthermore, the incorporation of constraints other than simple bounds and the tuning of parameters in such algorithms, e.g., the population size, are not always trivial [11]. Since in this paper the emphasis is on multi-objective dynamic optimization under uncertainty, which will cause that the computational effort increases even more, the latter category of methods is not applied [1].

The most basic scalarization approach to solve a multi-objective optimization problem is the weighted sum method [2], i.e., assigning a weight to each objective and writing the multi-objective optimization problem as a single objective optimization problem by taking a weighted sum of the different objectives. The main limitation of the weighted sum method is that Pareto points in non-convex regions cannot be calculated and it is scaling dependent. The NBI method does not have this problem and is therefore used in this work [7], [5]. In the NBI method, first the CHIM (*convex hull of individual minima*) is constructed, i.e., a plane containing all convex combinations of inidividual minima in the objective space. Then (quasi-)normal lines are constructed to this plane. The Pareto optimal points are defined as the intersection between the (quasi-)normal from any point on the CHIM and the boundary of the feasible objective space closest to the utopia point (i.e., the point containing the minima of all individual objectives). The rationale of the NBI method is to maximize the distance from a point on the CHIM along the (quasi-)normal through this point, without violating the original constraints [1], [5]. For a more detailed description of the frame of multi-objective dynamic optimization see, e.g., [11].

Normal Boundary Intersection (NBI) method

First some new terminology is introduced in order to explain the NBI method [7]. The *shadow minimum/utopia point*, $\boldsymbol{J}^*_{\boldsymbol{ut}}$, is defined as the vector containing the minima for each of the single objective functions. Note that the different values of the utopia point, are the different anchor points. The utopia point can only be attained in the rare case where one specific control vector $\mathbf{u}(t)$ minimizes all objective functions simultaneously, hence when all anchor points, J_i^* coincide. The *pay-off matrix*, $\boldsymbol{\Theta}$, contains the vectors of the utopia point towards the different anchor points.

The *convex hull of individual minima (CHIM)* consists of all convex combinations of the pay-off matrix $\mathbf{\Theta}$. Figure 1 displays the different concepts for a case with two objective functions. The red points J_1^* and J_2^* are the two different anchor points. The utopia point $\boldsymbol{J_{ut}^*}$ lies in the origin, point O. The CHIM is here represented by the dashed line connecting the two anchor points. The line underneath the CHIM connecting the two anchor points is the Pareto front.

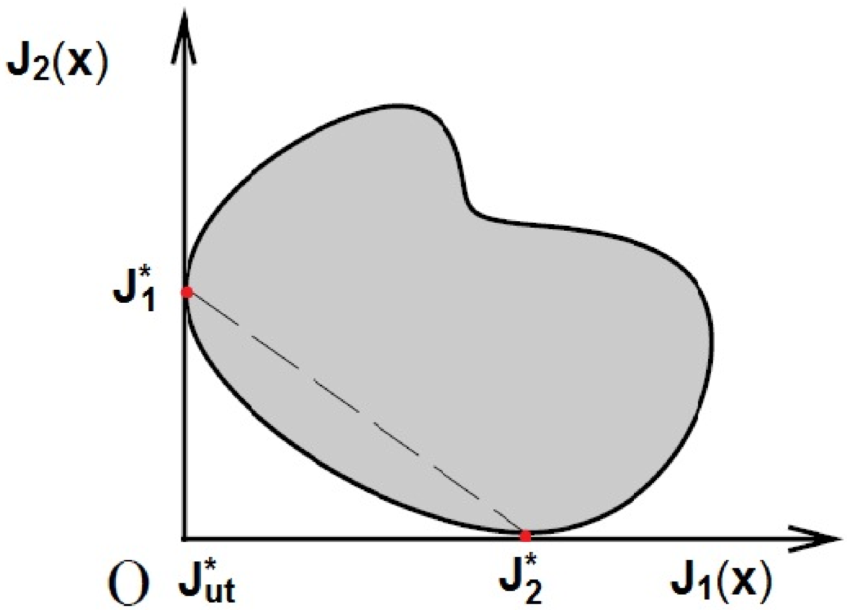

Figure 1: Representation of the utopia point, the anchor points and the CHIM for a bi-objective problem.

The NBI method, introduced by [7], is developed to overcome the deficiencies of the WS method and the ϵ-constraint method. The NBI method starts from the idea that the intersection point between the boundary of $J(\mathbf{x})$ and the normal vector, $\mathbf{n}$, in any point of the CHIM is a point on the Pareto front. If the intersection is present in a convex part of the boundary of $J(\mathbf{x})$, the obtained points are always Pareto optimal. Intersection points lying in the concave part of the function are obtained as well, which is both an advantage and a disadvantage. Because these points in the concave part are not guaranteed to be Pareto optimal, the NBI method may detect non-Pareto optimal points [1]. On the other hand, if the intersection points are Pareto optimal, they are also calculated by the NBI method, unlike the WS method. Figure 2 shows a graphical representation of the NBI method.

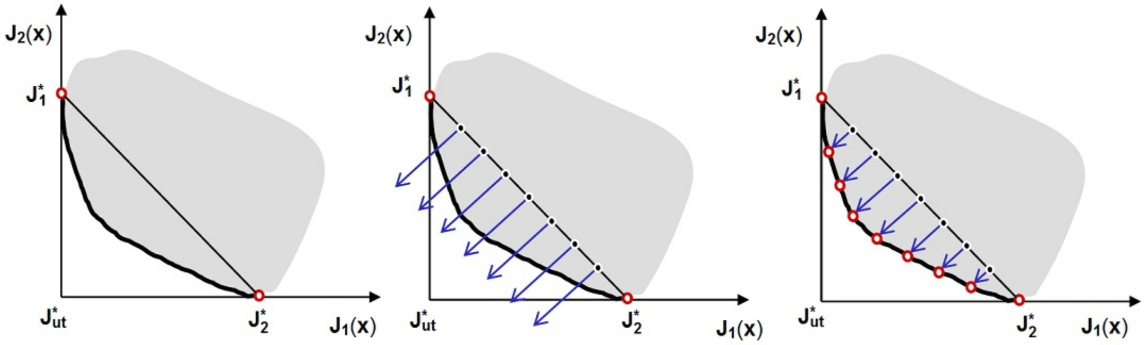

Figure 2: Representation of the NBI method for a bi-objective problem [1]

.

The mathematical formulation of the NBI method is a maximization problem. The distance d from the CHIM towards the utopia point is maximized while satisfying the constraints of the original optimization problem, Equations (3) and (4), and an extra vector constraint Equation (2) which ensures that the calculated point is a part of the feasible part of $J(\mathbf{x})$. Solving this problem is repeated for different combinations of $\boldsymbol{\beta}$. Das and Dennis [7] have proven that a uniformly distributed set of $\boldsymbol{\beta}$ gives rise to an evenly distributed set of Pareto points, which also can be seen in Figure 2.

$$\max_{\mathbf{x},d} \quad d \tag{1}$$

$$\text{s.t.:} \quad \boldsymbol{\Theta\beta} + d\mathbf{n} = \mathbf{J}(\mathbf{x}) \tag{2}$$

$$0 = \mathbf{h}(\mathbf{x}) \tag{3}$$

$$0 \geq \mathbf{g}(\mathbf{x}) \tag{4}$$

Numerical implementation and software

There is a large variety of techniques that can be used to solve dynamic optimization problems. In this work direct approaches are used that first discretize the optimal control problem into a nonlinear optimization problem (NLP), which can be solved afterwards with NLP solvers. The direct approaches can be divided in two types: *sequential methods* (e.g., single shooting [12], [13]) and *simultaneous methods* (e.g., multiple shooting [14], [15] and collocation [16]). The latter ones discretize both inputs and states while the former methods only discretize the inputs. Once the dynamic optimization problem is converted into a NLP, an appropriate NLP solver has to be chosen: this can be a stochastic optimization method (e.g., genetic algorithms, particle swarming optimization or simulated annealing) or a deterministic optimization method (e.g., interior point methods or sequential quadratic programming). Although stochastic methods have the major advantage that the objective function does not need to be differentiable (while it has to be in classic fast gradient-based optimization algorithms), there is not opted for the stochastic optimization methods. Usually a large amount of model and function evaluations is needed for stochastic optimization methods, which is a major disadvantage for large scale and highly constrained problems. Therefore a deterministic gradient-based optimization algorithm is selected.

It is chosen to discretize the problems using an orthogonal collocation discretization scheme. The rationale of orthogonal collocation is that the states and inputs are fully discretized with respect to time in finite elements. Per finite element there are four *collocation points* of which the first one is fixed and the three other ones should obey the model equations and are seen as equality constraints (i.e., so-called *collocation constraints*). Between each finite element there is also a constraint that ensures continuity (i.e., so-called *continuity constraints*). As interpolation between the *collocation points* a cubic Lagrange polynomial is used, with four collocation points situated at the Radau roots on each interval. State bounds are easily added in this technique. The fact that orthogonal collocation has hardly any problem with stiff systems is advantageous in case of numerically unstable systems.

The inhouse developed `Pomodoro` software [17] contains a collection of algorithms and tools for dynamic optimization and is implemented in `Python`. `Pomodoro` uses `CasADi` [18] as a backbone for the dynamic optimization problem formulation. `CasADi` is a software package for rapid prototyping of large-scale optimization problems with automatic differentiation using a symbolic/numeric approach. For solving the NLP, an interior point algorithm, `IPOPT` [19], has been used. For review purposes the `Pomodoro` software and the cited work [17], which has been submitted to Expert Systems with Applications, can be found on `http://www.student.kuleuven.be/~s0212066/pomodoro/`. For more information on the optimization methods and implementation, the reader is referred to Additional file 2 and [11].

Bibliography

[1] Logist, F., Houska, B., Diehl, M., Van Impe, J.: Robust multi-objective optimal control of uncertain (bio)chemical processes. Chemical engineering science **66**, 4670–4682 (2011)

[2] Das, I., Dennis, J.: A closer look at drawbacks of minimizing weighted sums of objectives for pareto set generation in multicriteria optimization problems. Structural Optimization (1997)

[3] Marler, T., Arora, J.: Survey of multi-objective optimization methods for engineering. Structural and Multidisciplinary Optimization **41**, 853–862 (2010)

[4] Haimes, Y., Lasdon, L., Wismer, D.: On a bicriterion of the problems of integrated system identification and system optimization. IEEE Transactions on Systems, Man, and Cybernetics **SMC-1**, 296–297 (1971)

[5] Logist, F., Van Impe, J.: Novel insights for multi-objective optimisation in engineering using normal boundary intersection and (enhanced) normalised normal constraint. Structural and Multidisciplinary Optimization (2012)

[6] Messac, A., Ismail-Yahaya, A., Mattson, C.A.: The normalized constraint method for generating the pareto frontier. Structural and Multidisciplinary Optimization (2003)

[7] Das, I., Dennis, J.: Normal-boundary intersection: a new method for generating the pareto surface in nonlinear multicriteria optimization problems. Siam Journal On Optimization (1998)

[8] Bhaskar, V., Gupta, S., Ray, A.: Applications of multi-objective optimization in chemical engineering. Reviews in Chemical Engineering **16**, 1–54 (2000)

[9] Reyes-Sierra, M., Coello, C.A.C.: Multi-objective particle swarm optimizers: A survey of the state-of-the-art. International Journal of Computational Intelligence Research (2006)

[10] Suman, B., Kumar, P.: A survey of simulated annealing as a tool for single and multiobjective optimization. Journal of the Operational Research Society (2006)

[11] Logist, F., Telen, D., Houska, B., Diehl, M., Van Impe, J.: Multi-objective optimal control of dynamic bioprocesses using acado toolkit. Bioprocess & Biosystems Engineering **36**, 151–164 (2013)

[12] Sargent, R.W.H., Sullivan, G.R.: The development of an efficient optimal control package. In: Optimization Techniques. Lecture Notes in Control and Information Sciences, vol. 7, pp. 158–168 (1978)

[13] Michalik, C., Hannemann, R., Marquardt, W.: Incremental single shooting — a robust method for the estimation of parameters in dynamical systems. Computers & Chemical Engineering **33**(7), 1298–1305 (2009)

[14] Leineweber, D.B., Schäfer, A., Bock, H.G., Schlöder, J.P.: An efficient multiple shooting based reduced {SQP} strategy for large-scale dynamic process optimization. part 1: theoretical aspects. Computers & Chemical Engineering " **27**(2), 157–166 (2003)

[15] Leineweber, D.B., Schäfer, A., Bock, H.G., Schlöder, J.P.: An efficient multiple shooting based reduced {SQP} strategy for large-scale dynamic process optimization: Part ii: Software aspects and applications. Computers & Chemical Engineering " **27**(2), 167–174 (2003)

[16] Biegler, L.T.: An overview of simultaneous strategies for dynamic optimization. Chemical Engineering and Processing: Process Intensification **46**(11), 1043–1053 (2007)

[17] Bhonsale, S.S., Vercammen, D., Vallerio, M., Hufkens, J., Cabianca, L., Nimmegeers, P., Telen, D., Logist, F., Van Impe, J.: Pomodoro - an open source toolkit for multiobject optimal control, and model based control and estimation. Preprint submitted to Expert Systems with Applications (2016)

[18] Andersson, J.: A General-Purpose Software Framework for Dynamic Optimization. PhD thesis, Arenberg Doctoral School, KU Leuven, Department of Electrical Engineering (ESAT/SCD) and Optimization in Engineering Center, Kasteelpark Arenberg 10, 3001-Heverlee, Belgium (October 2013)

[19] Wächter, A., Biegler, L.T.: On the implementation of an interior-point filter line-search algorithm for large-scale nonlinear programming. Mathematical Programming **106**, 25–57 (2006)
