# Supplementary material for: Dynamic optimization of biological networks under parametric uncertainty
Source: BMC Syst Biol. 2016 Aug 31;10:86. doi: 10.1186/s12918-016-0328-6 (PMC5006366; doi:10.1186/s12918-016-0328-6)
Supplement: Additional file 3 — Numerical results Case 1. A detailed overview of the numerical results for Case 1. (PDF 667 kb) [file 12918_2016_328_MOESM3_ESM.pdf]

Additional file 3: Numerical results Case 1

Philippe Nimmegeers, Dries Telen, Filip Logist, Jan Van Impe

Introduction

In this case study the terminal constraint expressing that the concentration of S_4 at time t_f should exceed 0.90 mM is robustified.

$$0.90 \text{ mM} \leq \mathbf{E}\left[S_4(t_\mathrm{f})\right] - \alpha_{S_4}\sqrt{\mathbf{Var}\left[S_4(t_\mathrm{f})\right]} \tag{1}$$

Since this constraint only looks at one bound that has to be exceeded, the 95% confidence region should be covered when a backoff parameter of $\alpha_{S_3} = 1.65$ is chosen. The intermediate accumulation objective function is not robustified for this case study (i.e., α_{J_2}).

For this case study the effect of assuming three uncertain parameters k_1, k_2 and k_3, characterized by a normal distribution (unless stated differently) with mean value 1 and 20 % relative standard deviation, is studied.

Parametric uncertainty distributions

In the polynomial chaos expansion approach, a priori information on the parametric uncertainty distribution can be taken directly into account via the orthogonal polynomials. Therefore it is investigated whether considering this a priori knowledge of the parametric uncertainty distribution for the robustified optimization provides a significant advantage. Two different parametric uncertainty distributions are studied: a normal parametric uncertainty distribution and a uniform parametric uncertainty distribution. As a remark, this should also be possible for the sigma points approach. However, the implementation is nontrivial and no references could be found in literature. For a normal parametric uncertainty distribution, standard (or normalized) Hermite polynomials are used as shown in Table 1.

Table 1: Normalized Hermite polynomials up till third order with ξ a standard normally distributed variable.

Order	Hermite	Roots
0	1	-
1	ξ	0
2	$\frac{1}{\sqrt{2}}(\xi^2 - 1)$	-1, $+1$
3	$\frac{1}{\sqrt{6}}(\xi^3 - 3\xi)$	$-\sqrt{3}$, 0, $\sqrt{3}$

For a uniform parametric uncertainty distribution, another type of polynomials is used. Assume that the parameters θ_{i_θ} are uniformly distributed with relative standard deviation $\sigma_{\theta_{i_\theta},\mathrm{rel}}$ and mean $\theta_{i_\theta,\mathrm{nom}}$. The interval $[\theta_{i_\theta,\,\min}, \theta_{i_\theta,\max}]$ covered by the uniform distribution is then computed as follows. Traditionally in literature, Legendre polynomials are chosen [1]. However, these Legendre polynomials only hold in the interval [0, 1]. Since the parameters considered in this work are not all in the interval [0, 1], it is chosen to derive orthogonal polynomials from the definition of orthogonal polynomials. For a uniform parametric uncertainty distribution the probability density function $\rho(\theta_{i_\theta}) = 1/\Delta\theta_{i_\theta}$, with $\Delta\theta i_\theta = \theta_{i_\theta,\max} - \theta_{i_\theta,\min}$.

The interval for the uniform parametric uncertainty distribution is assumed to be symmetric around the nominal parameter value $\theta_{i_\theta,\mathrm{nom}}$: $[\theta_{i_\theta,\mathrm{nom}} - \frac{\Delta\theta_{i_\theta}}{2}, \theta_{i_\theta,\mathrm{nom}} + \frac{\Delta\theta_{i_\theta}}{2}]$. Using the definition

of the variance in Equation (2), $\Delta\theta_{i_\theta}$ is derived:

$$\sigma^2_{\theta_{i_\theta}} = \frac{(\Delta\theta_{i_\theta})^2}{12}. \tag{2}$$

Hence, with $\sigma_{\theta_{i_\theta}} = \sigma_{\theta_{i_\theta},\mathrm{rel}}\theta_{i_\theta,\mathrm{nom}}$, this becomes:

$$\frac{\Delta\theta_{i_\theta}}{2} = \sqrt{3}\theta_{i_\theta,\mathrm{nom}}\sigma_{\theta_{i_\theta},\mathrm{rel}}. \tag{3}$$

Trade-off between end time and intermediate accumulation

The multi-objective optimization problem consists of minimizing the time needed to reach at least 0.90 mM of product S_4 and minimizing the accumulation of intermediates in the linear pathway. It is assumed that the final time t_f cannot exceed 10 s. In Figure 1 the Pareto fronts are shown for the linearization, sigma points approach, PCE1 and PCE2 approaches, respectively. It is already clear from the nominal problem that reducing the time needed to reach a level of 0.90 mM of S_4 leads to an increase in the intermediate accumulation and vice versa.

In Figure 1, there is a clear trend in the Pareto fronts that are receeding from the nominal optimal solution with an increasing backoff parameter value α_{S_4} (denoted by α in Figure 1). Both anchor points shift away from the nominal optimal solution. This is the price in performance (i.e., minimum intermediate accumulation) that has to be paid to ensure a minimum concentration of 0.90 mM for S_4. The performance in objective function values is worse than in the case of no parametric uncertainty, but in practice this uncertainty is present and should be taken into account.

In Figure 2 the Pareto fronts obtained with the linearization, sigma points, PCE1 and PCE2 are plotted together for $\alpha_{S_4} = 1.65$ and $\alpha_{S_4} = 1.96$, corresponding to levels of 5% and 2.5% constraint violations for a normal distribution. This allows to make a comparison between the different approximation techniques for uncertainty propagation for multi-objective dynamic optimization under uncertainty. A first observation for this case study shows that the linearization, sigma points and second order polynomial chaos expansion (PCE2) method take less backoff from the nominal solution than the first order polynomial chaos expansion approach (PCE1). The sigma points method and polynomial chaos expansion method use more sampling points, thus result in a better approximation.

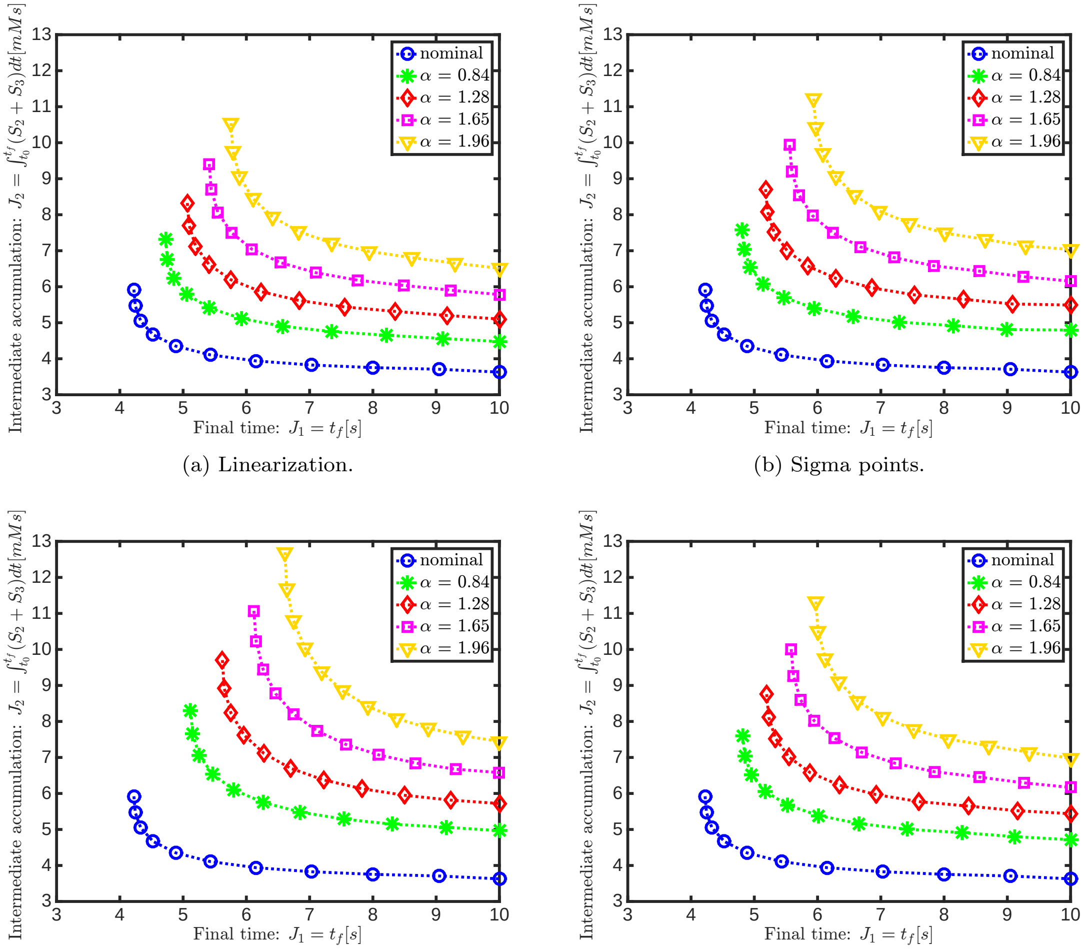

(a) Linearization.

(b) Sigma points.

(c) PCE1.

(d) PCE2.

Figure 1: Receeding Pareto fronts for Case 1 with increasing backoff parameter value α for the terminal constraint, obtained with the linearization (a), sigma points approach (b), PCE1 (c) and PCE2 (d) in case of 3 uncertain parameters k_1, k_2 and k_3.

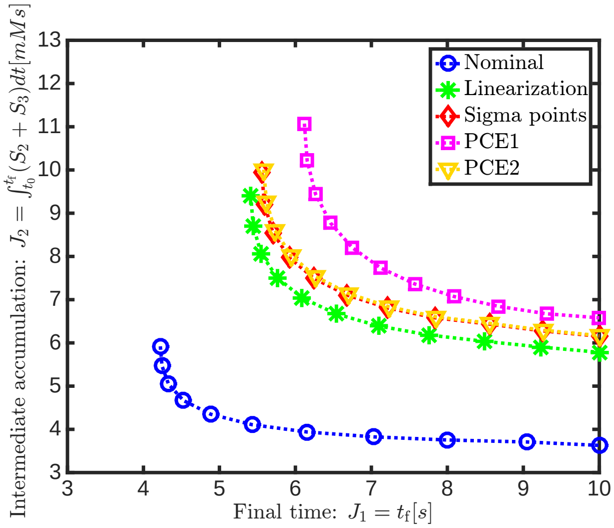

(a) Pareto front for $\alpha = 1.65$.

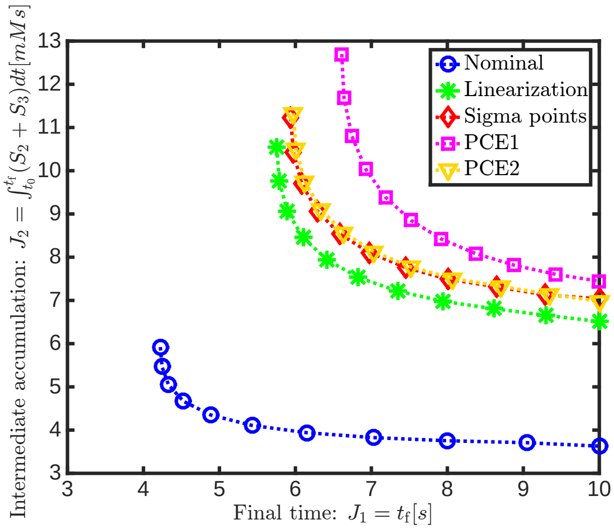

(b) Pareto front for $\alpha = 1.96$.

Figure 2: Comparison of Pareto fronts calculated with different approaches in case of 3 uncertain parameters k_1, k_2 and k_3: nominal (non-robustified), linearization, sigma points, PCE1 and PCE2 for $\alpha = 1.65$ (a) and $\alpha = 1.96$ (b).

A second observation in Figure 2 is that the Pareto fronts obtained with the sigma points approach and the second order polynomial chaos expansion method are nearly coinciding. This is expected, since the sigma points method and second order polynomial chaos approach are very similar in implementation. The sigma points are a subset of the *PCE sampling points* for the second order polynomial chaos expansion. This is shown in Table 2.

Table 2: Sampling points of the sigma points approach and PCE2 approach for Case 1 and a normally distributed parametric uncertainty distribution.

	Sigma points			PCE2		
	k_1 [mMs^{-1}]	k_2 [mMs^{-1}]	k_3 [mMs^{-1}]	k_1 [mMs^{-1}]	k_2 [mMs^{-1}]	k_3 [mMs^{-1}]
$\boldsymbol{\pi_0}$	1	1	1	1	1	1
$\boldsymbol{\pi_1}$	1	1	$1-\frac{\sqrt{3}}{5}$	1	1	$1-\frac{\sqrt{3}}{5}$
$\boldsymbol{\pi_2}$	1	$1-\frac{\sqrt{3}}{5}$	1	1	$1-\frac{\sqrt{3}}{5}$	1
$\boldsymbol{\pi_3}$	$1-\frac{\sqrt{3}}{5}$	1	1	$1-\frac{\sqrt{3}}{5}$	1	1
$\boldsymbol{\pi_4}$	1	1	$1+\frac{\sqrt{3}}{5}$	1	1	$1+\frac{\sqrt{3}}{5}$
$\boldsymbol{\pi_5}$	1	$1+\frac{\sqrt{3}}{5}$	1	1	$1+\frac{\sqrt{3}}{5}$	1
$\boldsymbol{\pi_6}$	$1+\frac{\sqrt{3}}{5}$	1	1	$1+\frac{\sqrt{3}}{5}$	1	1
$\boldsymbol{\pi_{7,\mathrm{PCE2}}}$				1	$1-\frac{\sqrt{3}}{5}$	$1-\frac{\sqrt{3}}{5}$
$\boldsymbol{\pi_{8,\mathrm{PCE2}}}$				$1-\frac{\sqrt{3}}{5}$	1	$1-\frac{\sqrt{3}}{5}$
$\boldsymbol{\pi_{9,\mathrm{PCE2}}}$				$1-\frac{\sqrt{3}}{5}$	$1-\frac{\sqrt{3}}{5}$	1

In case of a normal parametric uncertainty distribution, the expected value in the sigma points method is calculated in the same way as for the PCE2, but for the calculation of the variance the sampling points that are absent in the sigma points approach, are included. For the calculation of the expected value for the terminal constraint in the sigma points approach and PCE2 holds:

$$\bar{S}_{4,\mathrm{SP}} = \bar{S}_{4,\mathrm{PCE2}} = \frac{1}{6}\sum_{i=1}^{6} S_4(\boldsymbol{\pi_i}) \tag{4}$$

This motivates why the Pareto fronts of the sigma points and PCE2 are very similar.

Minimization of intermediate accumulation

In what follows the emphasis is on a single objective case, for instance one of the Pareto points. However, this analysis can be done for every point in the Pareto front and any level set. The single objective optimization of the intermediate accumulation is considered. It is assumed that the final time is fixed at 10 seconds. This analysis is done to investigate the approximation techniques for uncertainty propagation more in depth.

Computational aspects. A first aspect is the computational cost of including uncertainty in dynamic optimization. The number of required states and variables, together with the CPU time for the different approximation techniques for uncertainty propagation are presented in Table 3. The CPU times are presented for the largest backoff parameter used in this work, i.e., $\alpha_{S_4} = 1.96$, to give an upper bound on the computation times that are reached. The results in Table 3 confirm that taking uncertainty into account, leads to an increased computational time. An inherent property of the considered uncertainty propagation techniques, is the increase in number of states when the number of uncertain parameters increases. The increase in computational time for this case study is thus related to the increase in the size of the optimization problem. From this it is clear that the linearization and PCE1 approaches have a similar computation time and are the fastest of

the considered approximation techniques for uncertainty propagation, followed by the sigma points approach and PCE2 approach.

Table 3: Case 1 - Overview of the number of states, CPU time, expected values of the objective function, expected values of the terminal constraint and standard deviations for the different approximation techniques for uncertainty propagation when the enzymatic cost is minimized for $\alpha = 1.96$ for 3 uncertain parameters k_1, k_2 and k_3.

	Nominal	Linearization	Sigma Points	PCE1	PCE2
States	5	20	29	17	41
CPU time [s]	0.156	1.332	4.507	2.087	7.780
$\mathbf{E}[J]$	3.65	6.61	7.06	7.74	7.04
$\mathbf{E}[S_4]$	0.90	1.50	1.52	1.53	1.52
$\sqrt{\mathbf{Var}[S_4]}$	0	0.30	0.32	0.33	0.32

Control profiles. The determined optimal controls (i.e., the enzyme concentrations e_1, e_2 and e_3) with the approximation techniques for uncertainty propagation for $\alpha = 1.65$ are illustrated in Figure 3. It is concluded that qualitatively the control profiles look similar, but there is some backoff from the nominal optimal control. The sequential activation of the controls (i.e., the increasing and decreasing enzyme concentrations e_i) is qualitatively the same as the nominal activation. However, the peak values seem each time earlier in the robustified case than the nominal one, to ensure that the activation is sufficient and early enough.

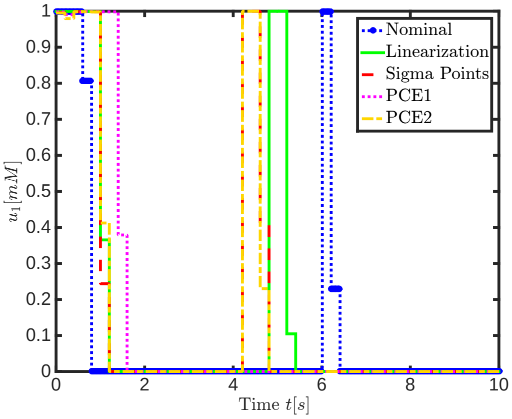

(a) Control e_1 (u_1).

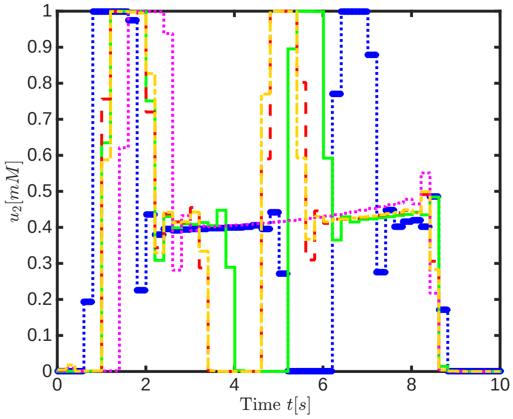

(b) Control e_2 (u_2).

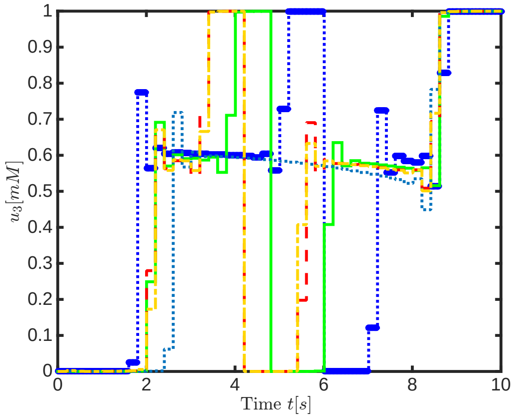

(c) Control e_3 (u_3).

Figure 3: Comparison of the control profiles e_1 (a), e_2 (b) and e_3 (c) calculated with linearization, sigma points approach, PCE1 and PCE2 for $\alpha = 1.65$ with the nominal control profile in case of 3 uncertain parameters k_1, k_2 and k_3.

General trends. As stated in the previous subsection on the multi-objective optimization under uncertainty, there are general trends observed between the different methods. These general trends (e.g., the similarities between the sigma points approach and PCE2) are investigated more in depth for the single objective optimization case.

First the expected value and 95% confidence bound of S_4 (based on $\alpha_{S_4} = 1.65$) are compared. This is done in Figure 4. From Figure 4 it can be seen that the expected state and 95% confidence bounds for S_4 are very similar when computed with the linearization, sigma points and PCE2 approaches. The PCE1 approach differs slightly in 95% confidence bound from the others. All lower bounds are at 0.90 mM as required by the imposed constraint in the implementation of the

approximation techniques for uncertainty propagation. The expected value is always above the required 0.90 mM. It should also be stressed that the time for which the bound of 0.90 mM for the expected state S_4 is reached, is between 5 and 6 seconds, which is substantially smaller than the fixed end time of 10 seconds.

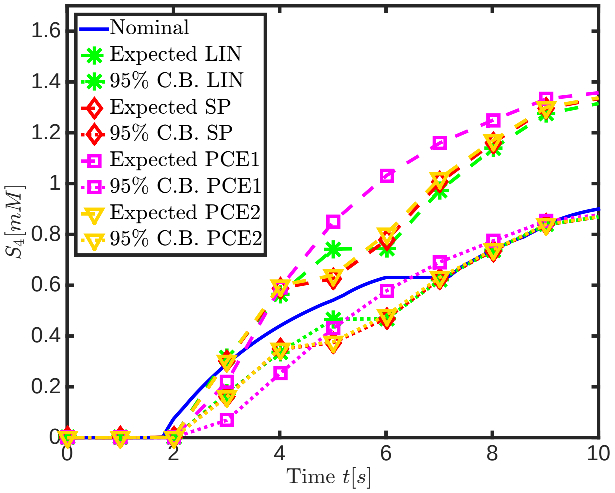

Figure 4: Comparison of the expected state S_4 and its 95% confidence bound calculated with linearization, sigma points, PCE1 and PCE2 with the nominal case ($\alpha = 1.65$) in case of 3 uncertain parameters k_1, k_2 and k_3.

Monte Carlo simulations

In order to investigate the performance of the different approximation techniques for uncertainty propagation with respect to the constraint violations, a Monte Carlo simulation with 1000 realizations (i.e., randomly generated parameter samples from the parametric uncertainty distribution) has been performed for the four approaches and is compared with the nominal case. The number of constraint violations, expected value of S_4 and the variance on S_4 for the different approaches and α_{S_4} are shown in Table 4.

Table 4: Case 1 - Results Monte Carlo simulations (N=1000) in case of three normally distributed uncertain parameters k_1, k_2 and k_3 for robustified terminal constraint with the number of constraint violations, mean terminal constraint values and variance on the terminal constraint.

	Nominal case	Linearization	Sigma points	PCE 1	PCE 2
$\boldsymbol{\alpha = 1.96}$					
$\bar{J}$	11.903	13.4600	13.600	13.829	13.583
σ_J	0.5578	0.9737	1.0087	1.016	0.9981
$\bar{S_4}$	0.88734	1.4739	1.5242	1.5500	1.5316
σ_{S_4}	0.1733	0.2878	0.2970	0.2993	0.2993
c_t violations	509 (50.9%)	30 (3.0%)	21 (2.1%)	20 (2.0%)	20 (2.0%)
$\boldsymbol{\alpha = 1.65}$					
$\bar{J}$	11.903	13.051	13.127	13.331	13.139
σ_J	0.5578	0.8624	0.8869	0.9049	0.8892
$\bar{S_4}$	0.8712	1.3357	1.3765	1.3910	1.3781
σ_{S_4}	0.1733	0.2614	0.2691	0.2683	0.2690
c_t violations	509 (50.9%)	51 (5.1%)	44 (4.4%)	39 (3.9%)	43 (4.3%)
$\boldsymbol{\alpha = 1.28}$					
$\bar{J}$	11.903	12.702	12.740	12.816	12.889
σ_J	0.5578	0.7728	0.7850	0.7975	0.8046
$\bar{S_4}$	0.8712	1.1994	1.2306	1.2493	1.2250
σ_{S_4}	0.1733	0.2342	0.2404	0.2420	0.2363
c_t violations	509 (50.9%)	101 (10.1 %)	87 (8.7 %)	**70 (7.0%)**	78 (7.8%)
$\boldsymbol{\alpha = 0.84}$					
$\bar{J}$	11.903	12.326	12.397	12.442	12.395
σ_J^2	0.5578	0.6753	0.6907	0.7003	0.6974
$\bar{S_4}$	0.8712	1.0698	1.0940	1.1095	1.0951
$\sigma_{S_4}^2$	0.1733	0.2095	0.2143	0.2154	0.2146
c_t violations	509 (50.9%)	199 (19.9%)	178 (17.8%)	164 (16.4%)	178 (17.8%)

From these simulations, it is observed that all four methods reduce the amount of constraint violations significantly: from 50.9% in the nominal case to even 2.0% for PCE2, when a backoff parameter value of $\alpha = 1.96$ is chosen.

In practice, the most interesting backoff parameter values are 1.65 and 1.96, corresponding to 5% and 2.5% violations in case of a normal distribution. In this case it is clear that the polynomial chaos expansion method of first order is superior in comparison with the linearization and sigma points approach when it comes to constraint violations. A lower backoff parameter value results into a too high number of constraint violations. The other α_i are computed to show the increase in constraint violations and the evolution of the variance and objective function when more backoff is introduced. From the results in Table 4 it is clear that the PCE1 method scores the best with respect to constraint violations, followed by PCE2 and sigma points. The performance of the sigma points method and the second order polynomial chaos expansion are, as shown throughout this case study, very similar.

If an uncertainty distribution is assumed for the constraint, the level of constraint violations should correspond exactly with the confidence level. A too low degree of violations is also not

wished, since the uncertainty is not propagated correctly in that case.

Furthermore, the expected values and variances of S_4 for a backoff parameter of $\alpha = 1.96$ from Table 3 which are predicted with the approximation techniques, are very close to the empirically calculated expected values and variances by Monte Carlo simulation in Table 4, For the sigma points, PCE1 and PCE2 approaches these predicted expected values and variances are an accurate estimation of the ones obtained by Monte Carlo simulation. For the linearization approach, this is not the case. However, the expected value of the intermediate accumulation objective function is not accurately predicted. This objective function is not robustified in this case study, as the variance on the objective function is not taken into account. Therefore, the predictions of the expected value of the objective functions are not accurate, when compared with a Monte Carlo setting.

Uniform distribution

Since prior knowledge on the parametric uncertainty distribution can be taken directly into account in the polynomial chaos expansion approach, the case of a uniformly distributed parametric uncertainty is studied with as expected value, the nominal parameter value and 20% relative standard deviation. The orthogonal polynomials are derived via the definition of orthogonal polynomials. The orthogonal polynomials and their roots for a uniformly distributed parameter $\theta_i \in [1-\sqrt{0.12}, 1+\sqrt{0.12}]$, are presented in Table 5.

Table 5: Case 1 - Derived orthogonal polynomials for uniform distribution up till third order for the parameter θ_i following a uniform parametric uncertainty distribution with expected value 1 and 20% relative standard deviation, which corresponds to the parameters k_1, k_2 and k_3.

Order	Polynomial	Roots
0	1	-
1	$\theta_i - 1$	1
2	$\theta_i^2 - 2x + \frac{24}{25}$	$\frac{4}{5}$, $\frac{6}{5}$
3	$\theta_i^3 - 3\theta_i^2 + \frac{366}{125}\theta_i - \frac{116}{125}$	$1 - \frac{3\sqrt{5}}{25}$, 1, $1 + \frac{3\sqrt{5}}{25}$

The *PCE sampling points* for the first and second order polynomial chaos expansion are determined by computing the roots of the second order polynomial and the roots of the third order polynomial, respectively.

Based on this table, it is clear that the *PCE sampling points* of PCE1 for a uniform parametric uncertainty distribution, are the same as those for a normal parametric uncertainty distribution. It can be proven for both case studies that the polynomial chaos expansion of first order is the same for a normal and uniform distribution parametric uncertainty distribution with the corresponding expected value $\theta_{i_\theta,\text{nom}}$ and relative standard deviation $\sigma_{i_\theta,\text{rel}}$.

A Monte Carlo simulation procedure with 1000 noise realizations from the uniform distribution has been followed and the results are summarized in Table 6.

Table 6: Case 1 - Results Monte Carlo simulations (N=1000) in case of three uniformly distributed uncertain parameters k_1,k_2 and k_3 for robustified terminal constraint with the number of constraint violations, mean values and variances on the objective function and terminal constraint, respectively.

	Nominal case	Linearization	Sigma points	PCE1	PCE2	PCE2 Uniform
$\boldsymbol{\alpha = 1.96}$						
$\bar{J}$	11.913	13.477	13.618	13.847	13.601	13.591
σ_J	0.5470	0.9601	0.9942	1.0000	0.9835	0.9790
$\bar{c_t}$	0.88917	1.4768	1.5273	1.5533	1.5347	1.5261
σ_{c_t}	0.1818	0.3015	0.3110	0.3132	0.3137	0.3118
c_t violation	516 (51.6%)	4 (0.4%)	1 (0.1%)	0 (0.0%)	1 (0.1%)	2 (0.2%)
$\boldsymbol{\alpha = 1.65}$						
$\bar{J}$	11.913	13.066	13.143	13.347	13.155	13.330
σ_J	0.5470	0.8489	0.8730	0.6813	0.8750	0.8881
$\bar{c_t}$	0.88917	1.3384	1.3792	1.3940	1.3809	1.3660
σ_{c_t}	0.1818	0.2740	0.2820	0.2808	0.2819	0.2754
c_t violation	516 (51.6%)	35 (3.5%)	22 (2.2%)	11 (1.1%)	22 (2.2%)	22 (2.2%)
$\boldsymbol{\alpha = 1.28}$						
$\bar{J}$	11.913	12.715	12.754	12.830	12.903	12.745
σ_J	0.5470	0.7601	0.7718	0.7824	0.7892	0.7707
$\bar{c_t}$	0.88917	1.2018	1.2331	1.2520	1.2276	1.2306
σ_{c_t}	0.1818	0.2455	0.2520	0.2534	0.2473	0.2512
c_t violation	516 (51.6%)	140 (14.0%)	117 (11.7%)	97 (9.7%)	113 (11.3%)	120 (12.0%)
$\boldsymbol{\alpha = 0.84}$						
$\bar{J}$	11.913	12.338	12.409	12.455	12.408	12.396
σ_J	0.5470	0.6637	0.6788	0.6865	0.6859	0.6864
$\bar{c_t}$	0.88917	1.0719	1.0962	1.1118	1.0973	1.0931
σ_{c_t}	0.1818	0.2197	0.2247	0.2257	0.2250	0.2222
c_t violation	516 (51.6%)	272 (27.2%)	242 (24.2%)	215 (21.5%)	240 (24.0%)	242 (24.2%)

Considering the constraint violations, all approaches do not exceed the the upper bound of 2.5% and 5% for backoff parameter values of 1.96 and 1.65 respectively. Based on Table 6 the second order polynomial chaos expansion for a normal parametric uncertainty distribution and a uniform parametric uncertainty distribution, can be compared in terms of performance. There is less backoff from the objective function when a uniform parametric uncertainty distribution is assumed, also the variance is reduced for the second order polynomial chaos expansion with respect to the assumption of a normal parametric uncertainty distribution. The percentage of constraint violations on the other hand is slightly higher when a uniform distribution is considered. However, for this case study, the difference in performance between a uniform parametric uncertainty distribution and a normal parametric uncertainty distribution for the polynomial chaos expansion, is small. Therefore, it should be stressed that including the additional information on the parametric uncertainty distribution can be useful. However, gathering information on the parametric uncertainty distribution is quite intensive and does not lead to a drastic improvement in performance for this case study.

Bibliography

[1] Poles, S., Lovison, A.: A polynomial chaos approach to robust multiobjective optimization. In: Deb, K., Greco, S., Miettinen, K., Zitzler, E. (eds.) Hybrid and Robust Approaches to Multiobjective Optimization. Dagstuhl Seminar Proceedings (2009)
