# Supplementary material for: Dynamic optimization of biological networks under parametric uncertainty
Source: BMC Syst Biol. 2016 Aug 31;10:86. doi: 10.1186/s12918-016-0328-6 (PMC5006366; doi:10.1186/s12918-016-0328-6)
Supplement: Additional file 4 — Numerical results Case 2. A detailed overview of the numerical results for Case 2. (PDF 708 kb) [file 12918_2016_328_MOESM4_ESM.pdf]

Additional file 4: Numerical results Case 2

Philippe Nimmegeers, Dries Telen, Filip Logist, Jan Van Impe

Case 2: Glycolysis based network with 1 output

The terminal constraint and enzymatic cost objective function are robustified in this case study as shown in following equations. This is done in order to reduce the variance on the objective function. In contrast to case study 1, this should allow to have a better prediction of the expected value and variance on the objective function.

$$0.675 \text{ mM} \leq \mathbf{E}\left[S_5(t_\mathrm{f})\right] - \alpha_{S_5}\sqrt{\mathbf{Var}\left[S_5(t_\mathrm{f})\right]} \tag{1}$$

$$J_2 = \mathbf{E}\left[x_\mathrm{extra}(t_\mathrm{f})\right] - \alpha_{x_\mathrm{extra}}\mathbf{Var}\left[x_\mathrm{extra}(t_\mathrm{f}))\right] \tag{2}$$

For simplicity, the backoff parameters α_{S_5} and $\alpha_{x_\mathrm{extra}}$ are assumed to be the same and are called α in the remainder of the text.

In this case study three parameters (k_cat, K_M and λ) are considered uncertain. First, the multi-objective optimization results are discussed, followed by a more in depth analysis of the minimization of the enzymatic cost. It is assumed that the parameters are normally distributed (unless stated differently) with as mean values their nominal parameter values and a relative standard deviation of 10%. Also the assumption of a uniform parametric uncertainty distribution is investigated.

Trade off between end time and enzymatic cost

The multi-objective optimization problem consists of minimizing the time needed to reach at least 0.675 mM of product S_5 and minimizing the enzymatic cost, i.e., the total enzyme consumption over the whole time span. In the robustified problem formulation, both the objective function for the enzymatic cost and the terminal constraint are robustified with backoff parameter α. It is assumed that the final time t_f cannot exceed 30 seconds. The two objectives, final time and enzymatic cost, are clearly conflicting: reducing the time needed to reach a level of 0.675 mM of S_5 leads to an increase in the enzymatic cost and vice versa.

Similar trends to those in Case 1 considering receeding Pareto fronts from the nominal optimal solution with increasing backoff parameter values can be observed in Figure 1: both anchor points shift away from the nominal optimal solution. This is the price in performance (i.e., minimum intermediate accumulation) that has to be paid to ensure a minimum concentration of 0.675 mM for S_5. However, it is also observed that the Pareto fronts change shape and range, when the backoff parameter increases. This is related to the feasibility of the Pareto points.

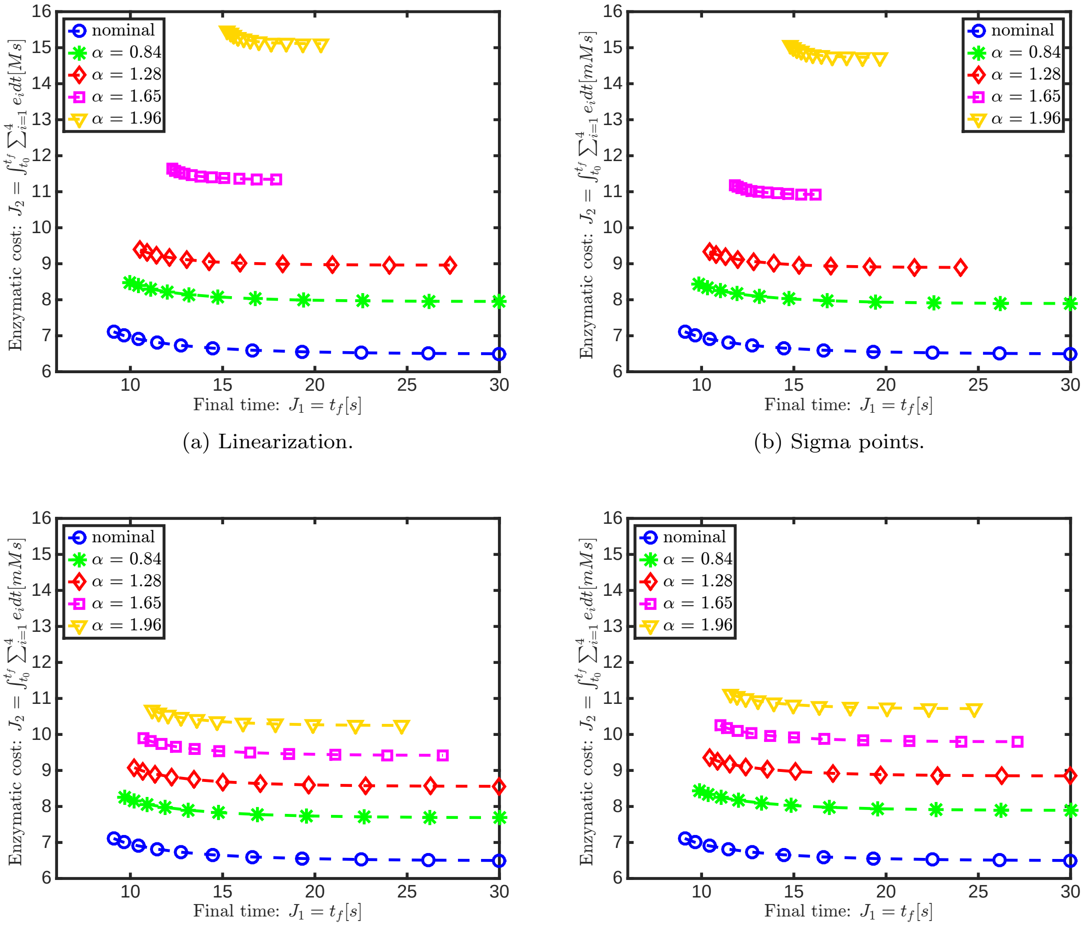

(a) Linearization.

(b) Sigma points.

(c) PCE1.

(d) PCE2.

Figure 1: Receeding Pareto fronts with increasing backoff parameter α for linearization (a), sigma points (b), first (c) and second order polynomial chaos expansion (d) approach in case of 3 uncertain parameters K_M, λ and k_{cat}.

The different approximation techniques for uncertainty propagation can be compared with Figure 2, in which the Pareto fronts obtained with the linearization, sigma points, PCE1 and PCE2 are plotted together for $\alpha_{S_4} = 1.65$ and $\alpha_{S_4} = 1.96$, corresponding to levels of 5% and 2.5% constraint violations for a normal distribution. A first observation for this case study shows that the linearization and sigma points approach take more backoff than the polynomial chaos approaches.

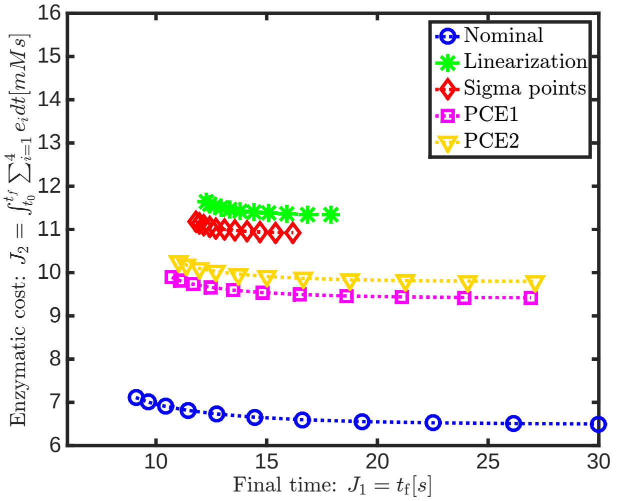

(a) Pareto front for $\alpha = 1.65$.

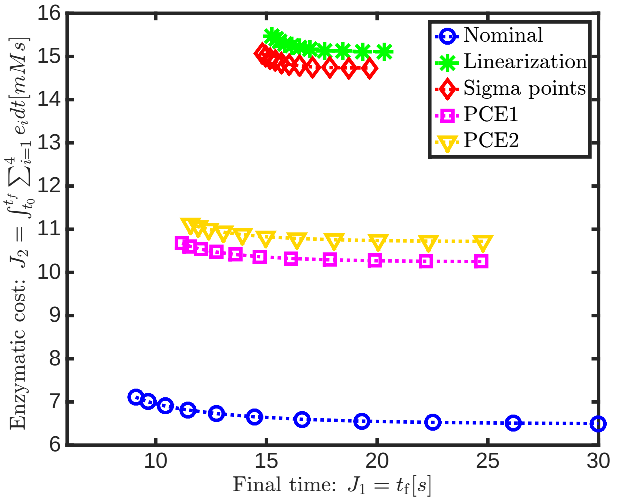

(b) Pareto front for $\alpha = 1.96$.

Figure 2: Comparison of Pareto fronts calculated with different approaches in case of 3 uncertain parameters K_M, λ and k_{cat}: nominal (non-robustified), linearization, sigma points, PCE1 and PCE2 for $\alpha = 1.65$ (a) and $\alpha = 1.96$ (b).

Secondly, when the backoff parameters decrease, it is observed that there is a similarity between the Pareto fronts for the PCE2 and sigma points approach as in Case 1. This can be explained from the variance that is taken less into account when the backoff parameter value decreases. Since the difference between the PCE2 and sigma points approach lies in the variance calculation, this explains the increasing difference in Pareto fronts with an increasing backoff parameter value. The

expected value calculation is the same for the sigma points and PCE2 approach in case of a normal parametric uncertainty distribution and has already been discussed in the previous case study. For this case study, the sigma points are also a subset of the PCE2 sampling points as shown in Table 1.

Table 1: Sampling points of the sigma points approach and PCE2 approach for Case 2 and a normally distributed parametric uncertainty distribution.

	Sigma points			PCE2		
	K_m [mM^{-1}]	λ [s^{-1}]	k_{cat} [$\text{mM}s^{-1}$]	K_m [mM^{-1}]	λ [s^{-1}]	k_{cat} [$\text{mM}s^{-1}$]
$\boldsymbol{\pi_0}$	1	0.5	1	1	0.5	1
$\boldsymbol{\pi_1}$	1	0.5	$1-\frac{\sqrt{3}}{10}$	1	0.5	$1-\frac{\sqrt{3}}{10}$
$\boldsymbol{\pi_2}$	1	$0.5-\frac{\sqrt{3}}{20}$	1	1	$0.5-\frac{\sqrt{3}}{20}$	1
$\boldsymbol{\pi_3}$	$1-\frac{\sqrt{3}}{10}$	0.5	1	$1-\frac{\sqrt{3}}{10}$	0.5	1
$\boldsymbol{\pi_4}$	1	0.5	$1+\frac{\sqrt{3}}{10}$	1	0.5	$1+\frac{\sqrt{3}}{10}$
$\boldsymbol{\pi_5}$	1	$0.5+\frac{\sqrt{3}}{20}$	1	1	$0.5+\frac{\sqrt{3}}{20}$	1
$\boldsymbol{\pi_6}$	$1+\frac{\sqrt{3}}{10}$	0.5	1	$1+\frac{\sqrt{3}}{10}$	0.5	1
$\boldsymbol{\pi_{7,\text{PCE2}}}$				1	$0.5-\frac{\sqrt{3}}{20}$	$1-\frac{\sqrt{3}}{10}$
$\boldsymbol{\pi_{8,\text{PCE2}}}$				$1-\frac{\sqrt{3}}{10}$	0.5	$1-\frac{\sqrt{3}}{10}$
$\boldsymbol{\pi_{9,\text{PCE2}}}$				$1-\frac{\sqrt{3}}{10}$	$0.5-\frac{\sqrt{3}}{20}$	1

Minimization of the enzymatic cost

For this case study, the single objective optimization of the enzymatic cost is considered. It is assumed that the final time is fixed at 30 seconds. This is done to investigate the approximation techniques for uncertainty propagation more in depth.

Computational aspects. In Table 2 an overview is of the number of states, the CPU time, objective function values, terminal constraint and their expected values and standard deviations for the different approximation techniques for uncertainty propagation for 3 uncertain parameters K_M, λ and k_{cat}. This is done for the largest backoff parameter value $\alpha = 1.96$ for the same reasons as mentioned in the first case study.

Table 2: Case 2 - Overview of the number of states, CPU time, objective function values, terminal constraint values and their expected values and standard deviations for the different approximation techniques for uncertainty propagation when the enzymatic cost is minimized for $\alpha = 1.96$ for 3 uncertain parameters (K_m, λ and k_{cat}).

	Nominal	Linearization	Sigma points	PCE1	PCE2
States	9	36	63	36	90
CPU time [s]	0.547	117.474	25.65	4.574	25.847
$\mathbf{E}[J]$	6.500	12.945	8.338	8.799	9.168
$\sqrt{\mathbf{Var}[J]}$	0	1.113	0.697	0.739	0.791
c_t	0.675	0.675	0.675	0.675	0.675
$\mathbf{E}[c_t]$	0.675	1.000	1.067	1.163	1.210
$\sqrt{\mathbf{Var}[c_t]}$	0	0.166	0.200	0.249	0.273

From this table, similar observations are made as in case study 1. However, the linearization approach is computationally the most expensive in this case study. While in case study 1, the increase in computational time is related to the increase in the size of the optimization problem, this cannot be the explanation for why the linearization approach takes the most CPU time. One explanation for the long CPU time of the linearization approach, can be the nonlinearity of the model in case study 2 and solving the sensitivity equations. The interconnection of the states in the sensitivity equations, makes the linearization approach computationally more challenging.

Control profiles. The optimal enzyme expression rates obtained for a backoff parameter value $\alpha = 1.65$ are shown in Figure 3. Qualitatively the control profiles look similar, but there is some backoff from the nominal optimal control. The sequential activation of the controls (i.e., the increasing and decreasing enzyme expression rates r_i) is qualitatively the same as the nominal activation. However for the enzyme expression rates r_2 and r_3, computed with the sigma points approach and linearization approach differ substantially from the nominal optimal control.

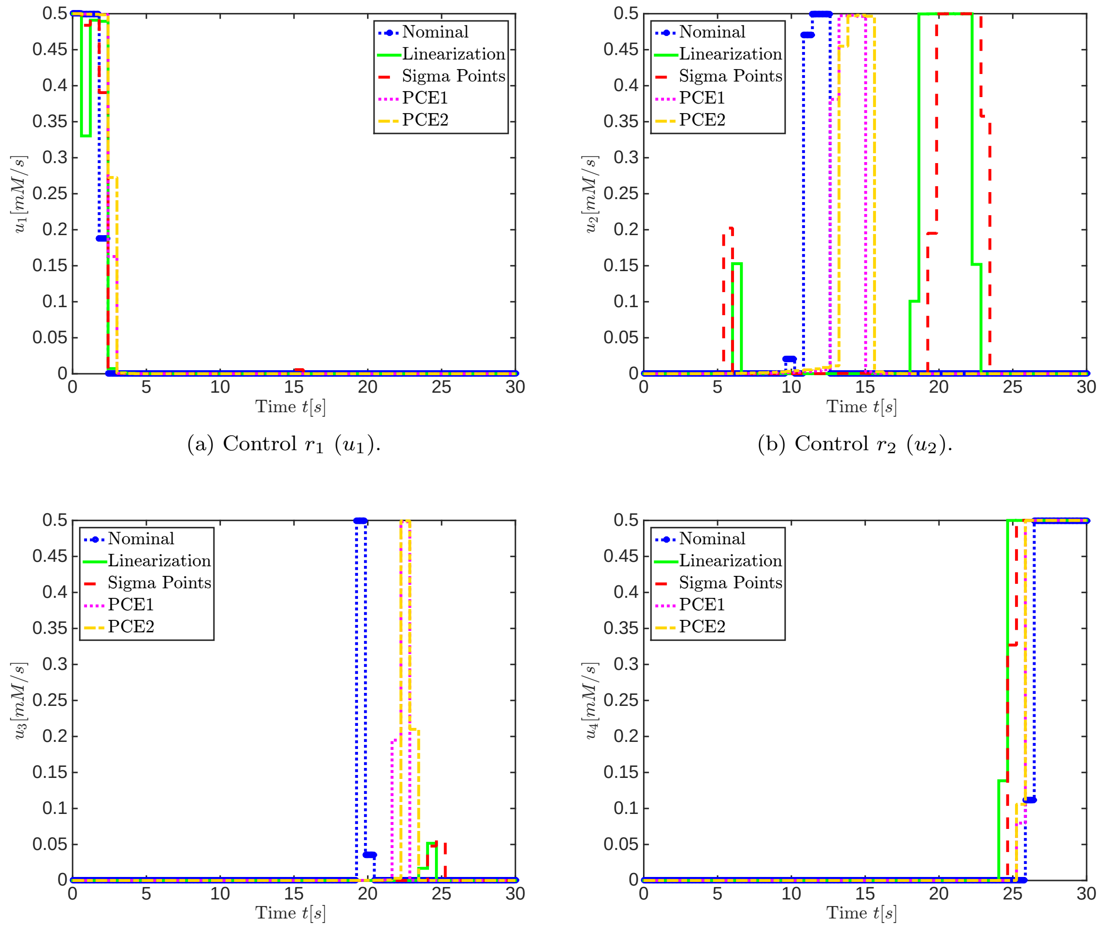

(a) Control r_1 (u_1).

(b) Control r_2 (u_2).

(c) Control r_3 (u_3).

(d) Control r_4 (u_4).

Figure 3: Comparison of the control profiles r_1 (a), r_2 (b), r_3 (c) and r_4 (d) calculated with linearization, sigma points approach, PCE1 and PCE2 for $\alpha = 1.65$ with the nominal control profile in case of 3 uncertain parameters K_M, λ and k_{cat}.

Predicted expected value and confidence bounds of terminal constraint. First the expected value and 95% confidence bound of S_5 (based on $\alpha_{S_{5)}} = 1.65$) are compared. This is done in Figure 4. From Figure 4 it can be seen that the expected state and 95% confidence bounds for S_4 are very similar when computed with the linearization, sigma points and PCE2 approaches. The PCE1 approach differs slightly in 95% confidence bound from the others. All 95% confidence bounds are at 0.675 at the end as required by the imposed constraint in the implementation of the approximation techniques for uncertainty propagation.

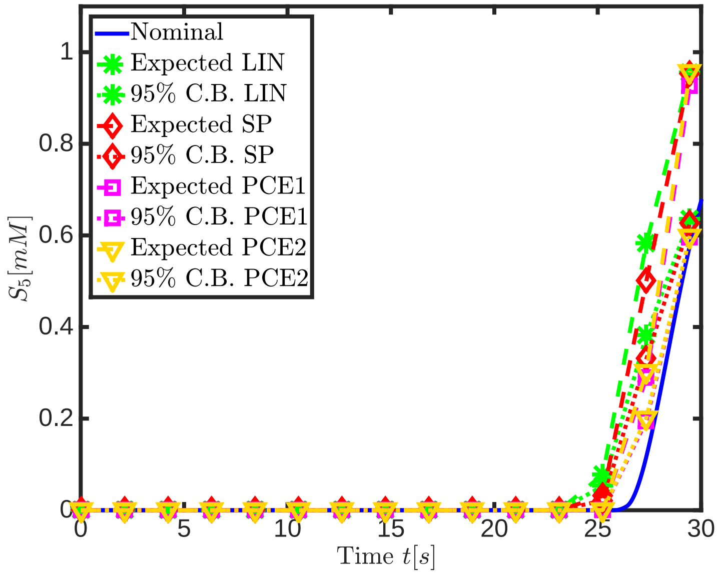

Figure 4: Comparison of the expected state S_5 and its 95% confidence bound calculated with linearization, sigma points, PCE1 and PCE2 with the nominal case ($\alpha = 1.65$) in case of 3 uncertain parameters K_M, λ and k_{cat}.

Monte Carlo simulations

The performance of the different approximation techniques for uncertainty propagation with respect to the constraint violations is investigated by performing a Monte Carlo simulation with 1000 noise realizations from a normal distribution. The number of constraint violations on S_5, mean values, and the variances for the objective function and terminal constraint, respectively are shown in Table 3 for the different approaches and backoff parameter values α_i.

Table 3: Case 2 - Results Monte Carlo simulations (N=1000) in case of three normally distributed uncertain parameters (K_m, λ and k_{cat}) for robustified terminal constraint and objective function with the number of constraint violations, mean values and variances on the objective function and terminal constraint, respectively.

	Nominal case	Linearization	Sigma points	PCE1	PCE2
$\boldsymbol{\alpha = 1.96}$					
$\bar{J}$	6.5268	12.996	12.549	8.7702	9.1358
σ_J	0.57543	1.1893	1.15230	0.78540	0.82107
$\bar{c}_t$	0.69319	1.0106	1.0106	1.1406	1.2099
σ_{c_t}	0.18291	0.17462	0.17752	0.27117	0.28089
c_t violation	477 (47.7%)	15 (1.5%)	18 (1.8%)	23 (2.3%)	13 (1.3%)
$\boldsymbol{\alpha = 1.65}$					
$\bar{J}$	6.5268	10.04	9.6011	8.2477	8.5514
σ_J	0.57543	0.88430	0.83741	0.73621	0.76632
$\bar{c}_t$	0.69319	1.0143	1.0157	1.0339	1.0933
σ_{c_t}	0.18291	0.20620	0.21414	0.25176	0.26120
c_t violation	477 (47.7%)	31 (3.1%)	39 (3.9%)	53 (5.3%)	34 (3.4%)
$\boldsymbol{\alpha = 1.28}$					
$\bar{J}$	6.5268	8.1255	7.9882	7.7085	7.9479
σ_J	0.57543	0.72752	0.71342	0.68546	0.70874
$\bar{c}_t$	0.69319	0.99866	0.97337	0.92494	0.97228
σ_{c_t}	0.18291	0.24727	0.24276	0.23126	0.23952
c_t violation	477 (47.7%)	78 (7.8%)	97 (9.7%)	135 (13.5%)	95 (9.5%)
$\boldsymbol{\alpha = 0.84}$					
$\bar{J}$	6.5268	7.4623	7.3437	7.1652	7.3409
σ_J	0.57543	0.66422	0.65172	0.63467	0.65110
$\bar{c}_t$	0.69319	0.87248	0.85006	0.81662	0.85081
σ_{c_t}	0.18291	0.22134	0.21712	0.20955	0.21593
c_t violation	477 (47.7%)	189 (18.9%)	217 (21.7%)	260 (26.0%)	213 (21.3%)

From these simulations, it is observed that all four methods reduce the amount of constraint violations significantly: from 47.7% in the nominal case to even 1.3% for PCE2, when a backoff parameter value of $\alpha = 1.96$ is chosen.

For this case study, the PCE2 method is superior in performance, when considering number of constraint violations.

Both PCE1 and PCE2 have a more accurate prediction of the expected value and variance of the objective function and terminal constraint than the linearization and sigma points approach, when comparing with the empirically calculated expected values and variances with Monte Carlo simulations.

Uniform distribution

For this case study, the integration of prior information on the parametric uncertainty distribution in the polynomial chaos expansion approaches is studied. The orthogonal polynomials for K_m, λ and k_{cat} are derived via the definition of orthogonal polynomials. Since K_m and k_{cat} follow a uniform distribution with expected value 1 and 0.1 as standard deviation in the interval $\left[1 - \frac{\sqrt{3}}{10}, \ 1 + \frac{\sqrt{3}}{10}\right]$ the same orthogonal polynomials are used. These are presented in Table 4.

For λ, that follows a uniform distribution with expected value 0.5, 0.05 relative standard deviation in the interval $\left[\frac{1}{2} - \frac{\sqrt{3}}{20}, \ \frac{1}{2} + \frac{\sqrt{3}}{20}\right]$ the orthogonal polynomials are described in Table 5. Also the roots of the higher order orthogonal polynomials are shown in Tables 4 and 5, since the *PCE sampling points* are determined by these.

Table 4: Case 2 - Derived orthogonal polynomials for uniform distribution up till third order for the parameter θ_i following a uniform parametric uncertainty distribution with expected value 1 and 10% relative standard deviation, corresponding to K_M and k_{cat}.

Order	Polynomial	Roots
0	1	-
1	$\theta_i - 1$	1
2	$\theta_i^2 - 2x + \frac{99}{100}$	$\frac{9}{10}, \frac{11}{10}$
3	$\theta_i^3 - 3\theta_i^2 + \frac{1491}{500}\theta_i - \frac{491}{500}$	$1 - \frac{3\sqrt{5}}{50}, 1, 1 + \frac{3\sqrt{5}}{50}$

Table 5: Case 2- Derived orthogonal polynomials for uniform distribution up till third order for the parameter θ_i following a uniform parametric uncertainty distribution with expected value 0.5 and 10% relative standard deviation, corresponding to λ.

Order	Polynomial	Roots
0	1	-
1	$\theta_i - \frac{1}{2}$	$\frac{1}{2}$
2	$\theta_i^2 - x + \frac{99}{400}$	$\frac{9}{20}, \frac{11}{20}$
3	$\theta_i^3 - \frac{3}{2}\theta_i^2 + \frac{1491}{2000}\theta_i - \frac{491}{4000}$	$\frac{1}{2} - \frac{3\sqrt{5}}{100}, \frac{1}{2}, \frac{1}{2} + \frac{3\sqrt{5}}{100}$

For a uniform parametric uncertainty distribution a Monte Carlo simulation procedure with 1000 noise realizations has been followed and the results are summarized in Table 6.

Table 6: Case 2- Results Monte Carlo simulations (N=1000) in case of three uniformly distributed uncertain parameters (K_m,λ and k_{cat}) for robustified terminal constraint and objective function with the number of constraint violations, mean values and variances on the objective function and terminal constraint, respectively.

	Nominal case	Linearization	Sigma points	PCE1	PCE2	PCE2 Uniform
$\boldsymbol{\alpha = 1.96}$						
$\bar{J}$	6.5517	13.048	12.599	8.8045	9.1717	9.1498
σ_J	0.5323	1.1015	1.0665	0.7270	0.7600	0.7600
$\bar{c}_t$	0.7023	1.0205	1.0208	1.1555	1.2254	1.2240
σ_{c_t}	0.1744	0.1659	0.1687	0.2590	0.2684	0.2690
c_t violation	470 (47.0%)	10 (1.0%)	13 (1.3%)	21 (2.1%)	7 (0.7%)	9 (0.9%)
$\boldsymbol{\alpha = 1.65}$						
$\bar{J}$	6.5517	10.079	9.6385	8.2798	8.5848	8.5683
σ_J	0.5323	0.8195	0.7762	0.6813	0.7092	0.7086
$\bar{c}_t$	0.7030	1.0259	1.0277	1.0476	1.1077	1.1056
σ_{c_t}	0.1744	0.1966	0.2042	0.2404	0.2495	0.2496
c_t violation	470 (47.0%)	28 (2.8%)	36 (3.6%)	55 (5.5%)	29 (2.9%)	29 (2.9%)
$\boldsymbol{\alpha = 1.28}$						
$\bar{J}$	6.5517	8.1573	8.0194	7.7383	7.9788	7.9676
σ_J	0.53234	0.6734	0.6603	0.6343	0.6559	0.6548
$\bar{c}_t$	0.70298	1.0121	0.98653	0.93751	0.98534	0.98319
σ_{c_t}	0.17443	0.2359	0.2316	0.2207	0.2287	0.2283
c_t violation	470 (47.0%)	73 (7.3%)	80 (8.0%)	118 (11.8%)	80 (8.0%)	80 (8.0%)
$\boldsymbol{\alpha = 0.84}$						
$\bar{J}$	6.5517	7.4912	7.3721	7.1928	7.3693	7.3622
σ_J	0.53234	0.6147	0.6031	0.5872	0.6025	0.6023
$\bar{c}_t$	0.70298	0.88444	0.8618	0.8279	0.8625	0.8613
σ_{c_t}	0.17443	0.2112	0.2071	0.1999	0.2060	0.2059
c_t violation	470 (47.0%)	163 (16.3%)	184 (18.4%)	224 (22.4%)	182 (18.2%)	183 (18.3%)

As in the first case study, the percentage of constraint violations is slightly higher when a uniform distribution is considered. For this case study, the difference in performance between a uniform parametric uncertainty distribution and a normal parametric uncertainty distribution for the polynomial chaos expansion, is small. Gathering information on the parametric uncertainty distribution from a parameter identification procedure is intensive and does not lead to a drastic improvement in performance for this case study.
